# Extraction and Analytical Methods for the Characterization of Polyphenols in Marine Microalgae: A Review

**DOI:** 10.3390/md22120538

**Published:** 2024-11-30

**Authors:** Gabriela Bermudez, Cristina Terenzi, Francesca Medri, Vincenza Andrisano, Serena Montanari

**Affiliations:** Department for Life Quality Studies, University of Bologna, Corso d’Augusto 237, 47921 Rimini, Italy; gabriela.bermudez2@unibo.it (G.B.); vincenza.andrisano@unibo.it (V.A.)

**Keywords:** marine microalgae, polyphenols, extraction, cell disruption, ultrasounds, HPLC, HPLC-MS, HPLC-Q-TOF, HPLC-MS/MS, spectrophotometric methods

## Abstract

Marine microalgae are emerging as promising sources of polyphenols, renowned for their health-promoting benefits. Recovering polyphenols from microalgae requires suitable treatment and extraction techniques to ensure their release from the biomass and analytical methodologies to assess their efficiency. This review provides a comprehensive comparison of traditional and cutting-edge extraction and analytical procedures applied for polyphenolic characterization in marine microalgae over the past 26 years, with a unique perspective on optimizing their recovery and identification. It addresses (I) cell disruption techniques, including bead milling, high-speed homogenization, pulsed electric field, ultrasonication, microwave, freeze-thawing, and enzymatic/chemical hydrolysis; (II) extraction techniques, such as solid–liquid extraction, ultrasound and microwave-assisted extraction, pressurized-liquid extraction, and supercritical CO_2_; (III) analytical methods, including total phenolic and flavonoid content assays and advanced chromatographic techniques like GC-MS, HPLC-DAD, and HPLC-MS. Key findings showed bead milling and chemical hydrolysis as effective cell disruption techniques, pressurized-liquid extraction and microwave-assisted extraction as promising efficient extraction methods, and HPLC-MS as the finest alternative for precise phenolic characterization. Unlike previous reviews, this study uniquely integrates both extractive and analytical approaches in one work, focusing exclusively on marine microalgae, a relatively underexplored area compared to freshwater species, offering actionable insights to guide future research and industrial applications.

## 1. Introduction

Microalgae comprises a highly heterogeneous group of photosynthetic microorganisms, able to grow in diverse environments; some are found in soils, but the vast majority are predominantly found in aquatic systems such as marine and freshwater [1]. Due to their fast growth rate and high biomass production compared to terrestrial plants, the exploitation of microalgae as “cell factories” has been gaining relevance over the years [2,3]. Moreover, these aquatic organisms represent a sustainable source for the recovery and extraction of a wide range of high-value molecules, such as proteins, polyunsaturated fatty acids (PUFAs), pigments (e.g., carotenoids), polysaccharides and antioxidants, that can be added to a variety of products, including food, cosmetics and nutraceuticals [1,2,4,5,6]. Among the more than 20,000 species forming part of the biodiverse world of microalgae [7], an important portion is broadly distributed in the marine ecosystems, being responsible for almost half of the global oxygen production [8]. Marine microalgae can exhibit a wide range of sizes and morphologies, including single cells, colonies, and extended filaments [6,8], requiring seawater, CO_2_, and sunlight to grow [8]. The ecological adaptation capacity of these organisms to high-salinity environments can modulate several metabolic pathways and the productivity/accumulation of different and unique secondary metabolites as a defense mechanism, especially of those with antioxidant properties in response to oxidative damage caused by salt stress [3,7]. Indeed, salinity has been proven to induce the production of high-value compounds, such as carotenoids and fatty acids [7]. Notable marine microalgae representatives include genera such as *Nannochloropsis*, *Tetraselmis*, *Dunaliella*, *Isochrysis*, and *Phaeodactylum* [6]. Examples of distinctive metabolites widely reported to be produced by marine microalgae with significant health-promoting benefits include carotenoids such as fucoxanthin obtained from *Phaeodactylum tricornutum* and β-carotene from *Dunaliella salina*, and the biologically active omega-3 PUFAs docosahexaenoic acid (DHA) and eicosapentaenoic acid (EPA) present in marine-derived oils recovered from *Tetraselmis* sp. and *Nannochloropsis oculata*, for instance [6]. In addition to carotenoids and PUFAs, another abundant group of secondary metabolites known for their significant antioxidant capacity are polyphenols [9], which are reported to be produced as a physiological adaptive response to stressful environments, allowing organisms to survive harsh conditions such as UV radiation, heavy metals, temperature, and salinity [9,10,11]. Research focused on the phenolic content in microalgae, including marine species, has notably increased in the past ten years [12].

**Figure 1:**
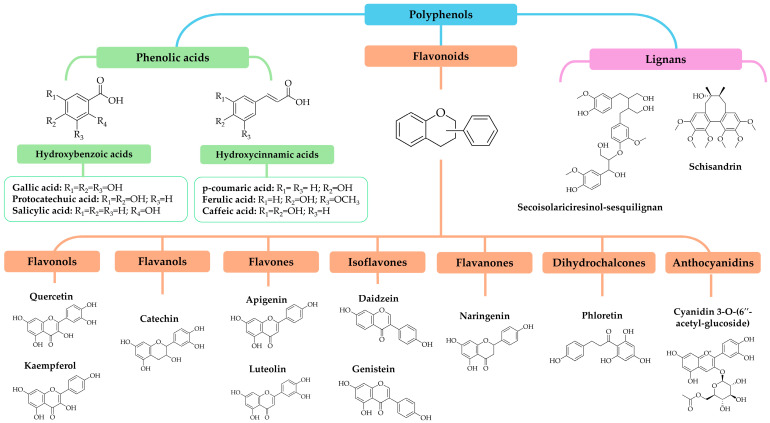
Principal classes of polyphenols reported in marine microalgae, including different phenolic acids (hydroxybenzoic and hydroxycinnamic acids), flavonoids, and lignans with their respective chemical structures.

Polyphenols are recognized as a group of phenolic systems with a basic structure ranging from single or double aromatic rings to more complex structures, bound with one or more hydroxyl groups (-OH) [13,14]. Based on structural differences, polyphenols can be classified into subclasses taking into consideration the number of rings in their structure and considering structural elements that are bound [14,15,16]. The main subclasses that have been found in marine microalgae include phenolic acids (such as hydroxybenzoic and hydroxycinnamic acids), flavonoids, and lignans [17,18,19,20]. Based on variations in their oxidation state and hydroxylation pattern, flavonoids are divided into a wider group that comprises flavonols, flavones, flavanols (also known as flavan-3-ols), flavanones, isoflavones, chalcones, anthocyanidins, and anthocyanins [14]. Figure 1 displays the chemical structures of the primary polyphenols identified in marine microalgae, categorized into their various subclasses. Further information regarding their distribution across some of the main species of marine microalgae can be found in Table 1. Additionally, based on the data collected in the different tables presented in this review, it is indicated that more than 40 types of polyphenols have been identified in marine microalgae.

Generally, these molecules are in the conjugated form with one or more sugar residues such as monosaccharides, disaccharides, or oligosaccharides [16]. The chemical structure of polyphenols has been related to strong antioxidant properties, contributing to free radical scavenging capacity [14]. Along with their antioxidant features, phenolic compounds can act as antiproliferative and anti-inflammatory agents, which has associated them with preventive effects against chronic diseases, including cardiovascular disease, certain cancers, neurodegenerative conditions, and metabolic disorders [15,16,21,22,23,24]. In addition, polyphenols can also be exploited for several technological applications, including the preservation of foods, the development of bioactive films, hydrogels and nanocomplexes, and acting as dyes and prebiotics [23,25,26,27].

Interestingly, various studies have demonstrated the potential to extract a greater quantity of phenolic compounds from marine microalgae than from freshwater *Chlorella* and *Spirulina* [11,28,29], encouraging future developments for the exploitation of these marine resources. For instance, when compared to *Chlorella vulgaris* and *Arthorspira platensis* methanol/water extracts from marine microalgae *Porphyridium purpureum* and *Nannochloropsis oculata* exhibited a higher content of polyphenols (flavan-3-ol compounds) that contributed to their superior bioactivity to manage metabolic disorders, supporting the potential use of these marine extracts as functional ingredients [29]. Similar results were observed in ethanolic extracts from *Chlorella*, which was found to contain the lowest phenolic content when compared to marine species *Nannochloropsis gaditana*, *Tetraselmis* sp., *Dunaliella* sp., *Phaoedactilum tricornutu* and *Navicula* sp. [28]. A more comprehensive study not only corroborated that marine microalgae such as *Phaeodactylum tricornutum*, *Nannochloropsis oculata*, *Isochrysis* sp., and *Tetraselmis* sp. could produce higher quantities of extractable polyphenols compared to *C. vulgaris*, but also demonstrated the impact of environmental conditions on intra-species variabilities in their phenolic content, suggesting that exploitation of these compounds could be optimized by selecting the proper cultivation and processing conditions, and that further determination of their phenolic identity is essential [11].

The extraction of phenolic compounds from some marine microalgae biomasses can entail unique challenges, due to the inherent characteristics of their cell walls, requiring the application of treatments and cell disruption techniques to enhance the release of these valuable compounds from the matrices, as in the case of the robust *Nannochloropsis* and *Chlorella* strains for instance [30,31]. To date, many methods have been described for the proper disruption of microalgae cell walls [31] as well as for the extraction of phenolic compounds [32]. Among the extractive techniques that can be applied, conventional extraction methods such as Soxhlet and solid–liquid extraction (SLE) have been well-established and widely used, paving the way for new discoveries and developments for polyphenol extraction. However, more efficient and greener assisted techniques have emerged to help overcome some of the big challenges of conventional extraction, as will be further discussed in this review.

Analytical characterization of the resulting phenolic-rich extracts from marine microalgae is an important aspect of evaluating the efficiency of the cell disruption and extraction techniques applied to samples. For this, popular spectrophotometric methods such as Folin–Ciocalteu to determine the Total Phenolic Content (TPC), and antioxidant assays focused on the ABTS^•+^ (2,2-azinobis-(3-ethylbenzothiazoline-6-sulfonate) and DPPH^•^ (2,2-diphenyl-1-picrylhydrazyl) radical scavenging methods have been widely employed [17,20,28,33,34,35,36]. However, high-performance chromatographic techniques combined with instrumental analysis are of great importance in assessing the real recovery of phenolic compounds in terms of their identity and quantities extracted. High-performance liquid chromatography (HPLC) is most frequently applied for this purpose, although some authors have also reported gas chromatography (GC), for the determination of polyphenols [37,38,39,40].

Given the complexity of some marine microalgae matrices [30,31] and the impact of cultivation conditions on the modulation of their phenolic composition [3,11,28], this review provides a comprehensive guide comparing different traditional and cutting-edge extraction and analytical methodologies that have been applied for the characterization of polyphenols in marine microalgae over the past 26 years, with a unique perspective on optimizing their recovery rates and identification. It addresses all stages, from sample preparation following microalgae cultivation to the most effective and efficient cell disruption, extraction techniques, and analytical methods reported, highlighting the advantages and disadvantages of each approach. Substantial progress has been made in the time frame covered by this review, with a growing number of key studies emerging from 2012 to 2024, shaping this field of research. Unlike previous reviews, this study uniquely integrates both extractive and analytical approaches in one comprehensive work, focusing exclusively on marine microalgae, an area that has been relatively unexplored compared to freshwater species, offering actionable insights to guide future research and industrial applications of these marine-derived bioactive compounds that are yet to be further exploited.

## 2. Recovery of Phenolic Compounds from Marine Microalgae

**Figure 2:**
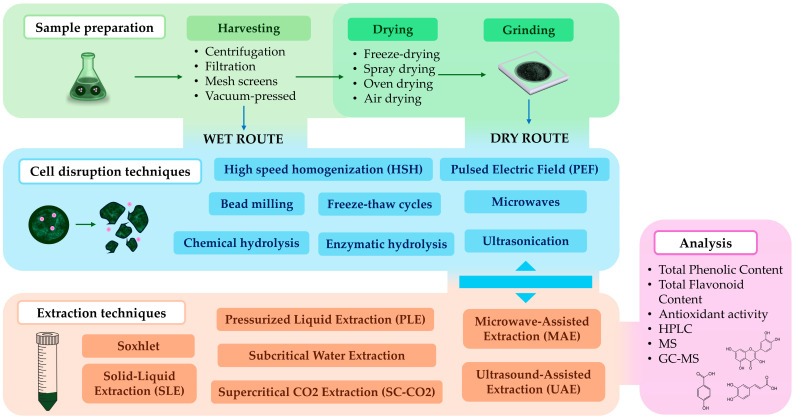
Overview of the different sample preparation, cell disruption, and extraction techniques and analytical methodologies applied to marine microalgae discussed in this review.

In microalgae, phenolic compounds mainly exist as intracellular metabolites [41,42]. Thus, their recovery often requires applying sample treatments aimed at disrupting cells to facilitate the release of compounds from the biomass during the extraction process [41]. Selecting a suitable treatment and extractive technique is crucial not only to ensure the extractability of these targeted compounds into the medium but also to preserve them throughout the process [32]. Several steps must be considered to efficiently recover polyphenols from microalgae, including sample preparation, cell disruption treatment, extraction technique, and solvent choice (Figure 2). Each of them are reviewed in the next sections, focusing on the operations and techniques that have been reportedly applied to marine microalgae.

### 2.1. Sample Preparation

Once microalgae are grown under optimal conditions, several steps are required to obtain the biomass for analysis. Sample preparation generally involves operations such as harvesting, drying, and grinding.

#### 2.1.1. Harvesting

During harvesting, the microalgae biomass is collected and separated from the cultivation media, in a process known as dewatering [43]. This is most commonly conducted by applying centrifugation to cell suspensions [18,36,44,45,46,47,48], but also filtration [20], mesh screens [49,50] or vacuum-pressed [51] has been reported for this purpose. Once harvested, the collected biomass could be immediately subjected to drying [29] or a previous washing step could be performed to remove the remaining salts [44,45,48,52,53].

#### 2.1.2. Drying and Grinding

Drying procedures applied on microalgae biomass include freeze-drying (lyophilization), spray drying, oven drying, sun drying, air drying, and microwave drying [43].

Freeze-drying is the most widely used drying technique employed to date for polyphenol extraction from marine microalgae [11,18,20,29,33,34,36,44,54,55], while spray drying [52] and air or oven-drying [56,57] have also been reported.

The choice of drying method and conditions is a crucial factor to consider since it can greatly affect the microalgae composition, including the phenolic content recovered [42,43,58,59,60]. Under the process of drying, polyphenols can degrade due to their heat sensitivity, the reason why techniques that offer control over the temperature or avoid thermal degradation are preferred [59]. For instance, Madhubalaji et al. [60] demonstrated how five different drying techniques modified the extraction rate of microalgae polyphenols, highlighting the effect of temperature on the results. Drying at low temperatures by freeze-drying (−110 °C) and sun-drying (30 °C) allowed the maximum recovery of polyphenols, followed by oven drying (60 °C), while temperature-intensive drum drying (120 °C) and spray-drying (100–150 °C) accounted for the loss of more than 50% of the phenolic content. Freeze-drying was considered the most appropriate method to preserve phenolic compounds, but its use might be limited by its higher energy consumption and operational costs [60].

During freeze-drying, water is removed from the frozen material by sublimation, at low pressure (around 1 kPa) and low temperature (less than −40 °C) [61]. Due to the low temperatures used, this procedure helps preserve thermolabile bioactive compounds [62]. This drying technique can also exert a mechanical effect promoting cell disruption. By gradually freezing the microalgae biomass, ice crystals form inside the cells, which can expand and create pores in the cell walls [61,62,63]. However, freeze-drying on its own may not be enough to disrupt microalgae cell walls completely, which is why it should be combined with cell-disruption techniques to increase the recovery of polyphenols [62,64].

Once dried, the solid biomass can be subjected to a grinding process to obtain a fine powder suitable for extraction purposes, which is typically achieved by using a mortar and a pestle [11,18,53].

#### 2.1.3. Biomass Processing (Dry and Wet Routes)

Although the vast majority of authors have subjected the microalgae biomass to the steps previously described to obtain a dry microalgae powder for extraction (known as “dry route”), some authors have omitted the drying process and instead have diluted the freshly harvested biomass/paste into suspensions with known concentrations [48,65,66,67,68,69,70] (known as “wet route”) followed by pre-treatment and extraction of polyphenols.

Choosing one or another route for extraction (dry or wet) depends on several factors. For instance, a drying procedure is normally performed to ensure the overall stability of the sample over time, preserve its chemical composition, ease subsequential processing handling and efficiency, and/or facilitate extraction [43]. However, drying is an energy-intensive step [71,72], and an efficient drying system must be carefully selected to avoid the thermal degradation of phenolic compounds [42,43]. On the contrary, the wet route tends to require less energy consumption by omitting the drying step and it has been associated with the recovery of high-value products compared to the dry route [30,71,73]. Nonetheless, a pre-treatment step of the wet biomass/suspension for cell disruption is needed to increase the efficiency of the extraction [71,74].

### 2.2. Treatment of Marine Microalgae Samples: Cell Disruption Techniques

Varying levels of complexity characterize microalgae cell walls. This ranges from a simple cell membrane to multi-layered arrangements of intra and extracellular material [61]. In this sense, morphological features can greatly impact the release of intracellular bioactive compounds from microalgae biomass, including polyphenols [42,62]. As a result, cell disruption techniques are often required to break down cell wall components and enhance solvent penetration and the overall efficiency of the extraction [31].

The cell walls of microalgae can be divided into four types based on their surface complexity [61]. Type 1 includes microalgae with simple and fragile cell membranes, such as *Dunaliella* and *Isochrysis*. Other marine microalgae such as *Porphyridium purpureum*, *Phaeodactylum tricornutum*, *Tetraselmis*, *Chlorella minutissima*, and *Nannochloropsis* sp. can be classified as Type 2, with different levels of extracellular material and cell wall complexity, making the extraction of intracellular compounds more challenging [61]. Table 2 outlines the primary representatives of marine microalgae along with their respective cell wall compositions based on increasing levels of complexity and cell disruption difficulty [31,75].

Cell disruption methodologies can be broadly classified as either mechanical or non-mechanical. This segment aims to show the main treatment methods that have been reported in the literature to date, concerning the documented or potential recovery of polyphenols from marine microalgae.

#### 2.2.1. Mechanical Treatment Techniques

Mechanical destruction of the microalgal cell wall occurs by applying physical forces on the sample, such as shear forces, waves, currents or thermal energy [31,61,85]. Mechanical techniques account for a non-specific disruption since they do not rely on the chemical composition of the cell walls, which makes them applicable to process all kinds of microalgae [31,61]. Some techniques are simple and easy to operate, while others require acquiring high-end and high-energy-consuming equipment [31]. The main mechanical cell disruption techniques applied on marine microalgae for the recovery of phenolic compounds are depicted in Table 3.

##### Bead Milling

During bead milling, small beads are responsible for grinding and dispersing the biomass into micro or nano-size [71]. The cell wall disruption occurs by the kinetic energy applied to force the collision between the beads and the microalgae cells, while the grinding chamber rotates [61,71]. The effectiveness of the cell lysis depends on several parameters, namely bead load, size and type (ceramic, zirconium, glass, plastic, steel), agitation speed, bead-milling chamber dimensions, cell concentration, and suspension flow rate [62,71]. This method leads to high-quality results while the energy required for processing is relatively low, even compared to other techniques [65,71].

Some authors have studied the disruption efficiency of bead milling on different marine microalgae species to improve the release of polyphenols. Sørensen et al. [86] reported an enhanced extractability of several phenolic compounds when bead milling was applied on *Phaeodactylum tricornutum* and *Tetraselmis chui*. Results showed a positive correlation between the degree of the bead mill treatment (measured as % of cell disruption) and the TPC release. Their study found that TPC values for microalgae *Phaeodactylum tricornutum* increased from 6.97 (0% cell disruption) to 17.19 mg GAE/g DW when cell disruption was equal to or higher than 48%, and that of *Tetraselmis chui* from ≈20 (0% disruption) to ≈30 mg GAE/g DW (from 60% disruption). The influence of bead milling on the recovery of individual phenolic compounds was also assessed, where cinnamic acid concentration was five times higher for *Phaeodactylum. tricornutum* at 65% disruption, and that of capsaicin, cinnamic acid, and dihydro-p-coumaric acid doubled or tripled for *Tetraselmis chui* at 99% cell disruption. Determining the optimal degree of cell disruption during bead milling is essential to obtain the highest polyphenol recovery from the biomass, which may vary for different microalgae species. Milling with ceramic beads was also reported for cell disruption of marine microalgae *Nannochloropsis* sp., achieving a maximum phenolic recovery yielding a TPC value of 14.9 ± 0.8 μg/mg DW [68].

##### High-Speed Homogenization (HSH)

High-speed homogenization (HSH) is based on the use of a homogenizer equipped with a stator-rotor assembly, normally made of stainless steel, whose design may vary keeping a small gap between both rotor and stator (100–3000 µm) [71]. Cell disruption occurs by hydrodynamic cavitation induced by high-speed stirring (10,000–20,000 rpm), where solid–liquid shear forces take place to break the microalgae cell walls [71,85,90]. In this sense, the speed (rpm) but also the treatment times are two critical parameters to optimize during experimental design. HSH has been applied on microalgae slurry and suspensions due to its simplicity and efficacy when relatively short processing times are required [61,71,85]. However, some disadvantages such as high energy consumption even at the lab scale [65], and aggressive disruption and shear-induced rise in temperatures, which is critical for thermolabile compounds, might limit its application [61,85].

As a pre-treatment to enhance the recovery of phenolic compounds, HSH has been employed to disrupt marine microalgae cells and enhance the recovery of phenolic compounds during solid–liquid extraction (SLE) [17,34]. The effectiveness of HSH in disrupting highly complex cell walls of distinct marine microalgae *Phaeodactylum tricornutum* and *Pavlova lutheri* was evaluated by Guedes et al. [87]. Results showed that HSH enabled the recovery of intracellular antioxidant molecules, surpassing other treatments such as ultrasound for this purpose.

##### Pulsed Electric Field (PEF)

In the Pulsed Electric Field (PEF) treatment, the microalgae suspension is positioned between two electrodes [61]. The cell walls are damaged, either reversibly or irreversibly, when short electric pulses (lasting from nanoseconds to milliseconds) of high intensity (voltages from 0.1 to 80 kV cm^−1^) are applied [31,71], which causes the formation of pores, known as “electroporation”, leading to cell permeabilization and release of intracellular compounds [31]. PEF is a novel microalgae cell disruption technique that has gained popularity in recent years [71]. However, PEF performance may vary depending on the microalgae under study, so species-specific method optimization is crucial to yield the highest recovery of bioactive compounds [70].

Parniakov et al. [48] found the pre-treatment with PEF (20 kV/cm, 1–4 ms, 400 pulses, 13.3–53.1 kJ/kg at 293 K) to improve the extractability of phenolic compounds from *Nannochloropsis* spp. suspensions (1%). Interestingly, the choice of solvent composition emerged as a critical factor, with greater recovery rates observed (1.5 to 2 times higher) when using a 50% binary mixture of DMSO or ethanol with water respectively. A similar study was conducted on *Phaeodactylum tricornutum* and *Tetraselmis chuii* by Kokkali et al. [70], which compared two different PEF pre-treatments, a first one of moderate intensity and short duration (3 kV/cm/45 pulses) and a second one of low intensity and long duration (1 kV/cm/400 pulses). The results indicated that the recovery of TPC for *Tetraselmis chuii* increased from 4.38 (control) to 6.42–6.70 mg GAE/g DW after PEF pre-treatment at 3 kV/cm and 4 h of SLE, regardless of the solvent used (DMSO 50% or water). However, for *Phaeodactylum tricornutum*, there was no significant influence of PEF pre-treatment conditions on the extractability of polyphenols, but there was an impact based on the solvent used instead.

##### Ultrasonication

Ultrasonication cell disruption occurs due to acoustic cavitation [85,91]. During this process, ultrasound waves (20 kHz to 1 MHz frequency) generate a successive series of high-pressure (compression) and low-pressure (decompression) cycles, with the subsequent formation of “microbubbles” that grow in the medium until they violently implode, producing shock waves known as “cavitation” [61,62,71,91]. The cavitation is responsible for both mechanical/physical (acoustic vibrations, inter-particle collisions and heat) and chemical effects (water thermolysis generating free radicals) for cell wall rupture [61,91]. The efficiency of this method depends on the intrinsic characteristics of the microalgae species, its concentration and the operational parameters involved such as time, frequency, acoustic power, temperature and number of cycles [61,92]. Temperature should be monitored and controlled during the treatment since heat production is inherent to the process [85].

To date, this pre-treatment technique has been applied to microalgae research mainly focused on biodiesel, bioethanol and biogas research field [61]. Thus, studies on ultrasonication as a pre-treatment step to enhance polyphenol recovery from microalgae are scarce. One study applied ultrasound pre-treatment using 20 kHz on *Chlorella* sp. samples to assist the recovery of polyphenols by enzymolysis, proving effective in enhancing TPC yields by 17%, 50% and 20% for hydrolysis times of 2, 4, and 6 h, respectively [88]. Most authors have employed ultrasound as the main extractive technique, regarded as Ultrasound-Assisted Extraction, as Section 3.3.1 further explores.

##### Microwave

Microwaves have also been employed to disrupt microalgae cell walls. These electromagnetic waves comprise frequencies between 0.3 and 300 GHz, of which 2.45 GHz is considered optimal for heating and cell disruption [62,85,90]. This mechanism is based on the dipole rotations generated when polar and dielectric compounds are subjected to the electromagnetic fields induced by the microwaves. This rotational movement causes a rapid and uniform distribution of heat energy into the biomass through friction [31,62]. Therefore, intracellular water evaporates, which increases the internal pressure forcing the cell walls to expand and break, releasing bioactive compounds [31,62,85]. For optimal results, parameters such as biomass concentration, treatment time and power of microwaves have to be evaluated [61]. Although microwave is a simple and rapid cell disruption process, some drawbacks to consider are the high temperature that may limit the recovery of labile compounds and the potential formation of free radicals [61].

Studies on microwaves as a pre-treatment step for recovering phenolic compounds from marine microalgae are also scarce. The superiority of microwave pre-treatment to increase the release of value-added compounds such as lipids [93,94] from marine microalgae has been the main focus of applying this pre-treatment technique, leading to positive results. Some studies conducted in non-marine microalgae could give some insights into the utility of this technology for pre-treatment purposes for future developments, which seem to depend on microalgae species and optimization of operational parameters. Microwaves were reported to induce a high cell disruption degree (94.92%) in marine microalgae *Nannochloropsis oculata* [30]. Microwave treatment (2450 MHz, 1400 W, 120 s) on *Spirulina* sp. led to the greatest release of phenolic compounds (41.90 mg GAE/g) when compared to the untreated sample (31.52 mg GAE/g) [42]. However, opposite results were observed for *Coelastrella* sp. suspensions since microwave cell disruption (32.03% at 800 W for 12 min) was the least effective method for releasing phenolic compounds, which was attributed to polymerization or oxidation of the present polyphenols due to temperature rise [65].

The use of microwave as an extractive methodology, known as Microwave-Assisted Extraction, rather than a pre-treatment step is further explored in Section 3.3.2.

##### Freeze–Thaw Cycles (FTCs)

Freeze–thaw cycles (FTCs) involve subjecting samples to consecutive cycles of freezing and defrosting, thus exposing them to high and low temperatures. As a result, cell disruption is obtained by the formation of ice crystals during the freezing step, followed by a cell expansion when temperatures increase (thawing step), enhancing cell permeabilization [61,95,96]. This method is characterized by its inherent simplicity, standing as an efficient and affordable technique [95]. Recovery of phenolic compounds from microalgae using FTCs has been documented for non-marine species such as *Spirulina platensis* [89] and *Coelastrella* sp. [65] with encouraging results for future marine-derived developments, yielding higher TPC and individual phenolic levels even compared to other treatments such as ultrasound, even if the cell disruption degree was low [65].

#### 2.2.2. Non-Mechanical Treatment Techniques

Disruption of microalgae cell walls by non-mechanical techniques could also be promoted by the interaction of chemicals or enzymatic agents with specific cell wall components, resulting in cell permeabilization [31].

Polyphenols in microalgae can be found in a free form readily soluble and easy to extract (free phenolics), or chemically linked to components in the cell walls (bound phenolics) requiring to be released by chemical or enzymatic hydrolysis [17], which renders non-mechanical treatments of high value in the recovery of phenolic compounds from these matrices.

##### Chemical Treatment

Among the chemical treatments, acid and alkali methods stand out, employing mainly strong acids such as H_2_SO_4_ and HCl and bases like NaOH. Acids act by degrading the polysaccharide matrix in cell walls (like cellulose) while alkali treatment can induce saponification and salvation reaction creating pores in the walls [97]. Acid and alkali treatments tend to be easily accessible, cheaper, and have a low energy consumption compared to mechanical ones [31,61], but low selectivity and degradation reactions have to be considered [61]. Among the operational parameters involved in the optimization of the method acid/base concentration, temperature, biomass solid–liquid ratio, and type of species can be mentioned [61]. A recent study conducted by Zhou et al. [17] evaluated the efficiency of alkaline–acid hydrolysis on the release of bound phenolic compounds from 7 different marine microalgae species before conventional SLE. Results showed a significant increase in the recovery of polyphenols, ranging from TPC levels of 1.83–6.45 mg GAE/g DW of free phenolics before treatment to 4.03–26.03 mg GAE/g DW after treatment (bound phenolics), and a favored release of the lignans secoisolariciresinol-sesquilignan and schisandrin after treatment. The alkaline–acid method applied by the authors, phenolic content, and profiling for each marine microalgae is described in Table 4.

##### Enzymatic Treatment

Enzymatic hydrolysis is considered a green pre-treatment technique due to the use of enzymes acting as natural catalysts able to break down specific cell wall components, depending on each specific enzymatic target [97,98]. In this sense, the enzymes used can comprise cellulases, proteases, lipases, glycosidases, amylases, xylanases, peptidases, and lipases [61]. Rather than using individual enzymes, a blend is often preferred to increase the hydrolysis efficiency [31]. Although its high selectivity, biological specificity and mild reaction conditions [98], the use of enzymes might result in high costs and long periods of incubation might be necessary [61]. Operational parameters to evaluate during the process involve temperature, pH, enzyme amount and time [61]. For instance, a blend of the enzymes cellulase and mannanase was found to alter drastically the cell morphology of marine microalgae *Nannochloropsis* sp., enhancing the potential release of intracellular material under optimal experimental conditions (53 °C, pH 4.4, 24 h) [83].

## 3. Extraction Techniques to Recover Polyphenols from Microalgae

After proper sample preparation and pre-treatment of the microalgae biomass, the separation of the phenolic compounds of interest from the solid matrix is achieved by applying extraction techniques with the help of suitable solvents [99]. Among the various extraction techniques, conventional methods, including solid–liquid extraction (SLE), are the most widely employed and reported to date [100] due to their inherent simplicity, cost-effectiveness, and extensive applicability [33]. Nevertheless, these methods are associated with certain limitations, including lower extraction efficiency, increased solvent volumes required, and time-intensive procedures [32]. Non-conventional novel techniques such as Ultrasound-Assisted Extraction (UAE), Microwave-Assisted Extraction (MAE), Pressurized-Assisted Extraction (PLE), and Supercritical-Fluid Extraction (SFE) have been developed as environmentally friendly alternatives to overcome the challenges related to the use of conventional techniques, offering improved yields while reducing time, energy, and solvent consumption [32,41,101].

Depending on the microalgae species, the ideal extraction method and solvent system may vary as their efficiency is highly influenced by the matrix properties as well [33]. For this reason, optimization studies are essential to determine the best conditions resulting in the highest extraction rates. This chapter aims to explain the main extraction techniques, their working mechanisms, and the solvents that have been utilized in the research of polyphenols from marine microalgae to date.

### 3.1. Solvent Choice

To maximize the extraction of phenolic compounds, the choice of the solvent in terms of nature and volume has to be established based on solubility criteria, since the solvent system has a major influence on the recovery of phenolic compounds from the matrix [33,102]. The phenolic nature of polyphenols renders them molecules with a relative hydrophilicity, and therefore soluble in solvents with higher polarity, such as methanol, ethanol, acetonitrile, ethyl acetate, and acetone, generally in binary mixtures with water [37,103]. However, due to the structural differences among all polyphenol subclasses, including their level of conjugation and the number of hydroxyl groups present, selecting a suitable solvent system should be carefully assessed to ensure the highest recovery of all phenolic forms [32]. For instance, more polar phenolic acids may be easily extractable with polar solvents like water, whereas less polar flavonoids may require a higher addition of organic solvents [104]. In this sense, the quantitative extraction of polyphenols entails an optimization of the extractive solvent based on the affinity with the targeted polyphenols, but also with the biomass [104].

Extraction of polyphenols from marine microalgae has mainly entailed the use of organic solvents such as methanol and ethanol and its binary mixtures with water [17,18,19,20,28,29,33,34,44,68,69,105,106]. Other organic solvents that have been reported include ethyl acetate [36,107], dimethyl sulfoxide (DMSO) [48,52,68], acetone [54], dichloromethane [55,108], hexane [34,36,55,109] and chloroform [55]. Among this group of solvents, some belong in the category of “generally recognized as safe” (GRAS), which include ethanol, water, and ethyl acetate [33,107], reportedly employed in the development of greener and novel extractive processes [105,107].

More recently, Deep Eutectic Solvents (DESs) have been proposed as eco-friendly alternatives proven to extract higher yields of polyphenols from microalgae compared to conventional solvents [110,111,112]. DESs systems tested include those based on mixtures of choline chloride with different polyols [110,111], organic acids and amides [112]. Another novel alternative that has been introduced as extractive systems of polyphenols from microalgae is the supramolecular solvents (SUPRAS) [113]. These systems represent promising solvent alternatives for future advances yet to be applied to marine species.

### 3.2. Conventional Methods

Conventional methods for extracting polyphenols from marine microalgae typically include Soxhlet extraction and SLE. While some studies have looked at Soxhlet extracts for their phenolic content and antioxidant properties [57,108], most studies to date have employed conventional SLE, which are displayed in Table 5. The extraction parameters used and the total TPC values obtained by several authors on the recovery of polyphenols from marine microalgae are described.

In SLE, organic solvents are added directly to the samples, and the efficiency of the extraction will depend on experimental factors such as solvent choice, extraction time, and temperature [100]. During extraction, agitation is a common procedure, normally assisted by the use of shaking incubators [17,33] or magnetic stirrers [114]. Although higher working temperatures may favor the extraction efficiency of polyphenols [33], most studies on marine microalgae have been conducted at room temperature (RT), as shown in Table 5.

Regarding extraction times, it is well known that conventional techniques are time-consuming, which is one of the main limitations of their application [32]. Table 5 shows that the extraction times required for SLE extraction of polyphenols from marine microalgae range from a few hours to days. This, together with the lower efficiency associated with these methods [32], reinforces the need to develop faster approaches, such as the assisted non-conventional approaches discussed in the next paragraph.

To improve the efficiency of the extraction by SLE, some authors have applied cell disruption pre-treatment techniques on the biomasses studied to ease the release of phenolic compounds, including HSH (rotor-stator homogenizers) [17,34], chemical alkaline–acid hydrolysis [17], bead milling [69], and PEF [48,70], which have already been described in Section 2.2.1.

### 3.3. Unconventional Methods: Green Techniques

These novel methods have the advantage that they already involve a cell disruption technology, so pre-treatments or not normally employed as compared to SLE. Assisting the extraction process by applying cell disruption mechanisms already discussed in Section 2.2.

**Table 5:** Solid–liquid extraction (SLE) methods applied for the recovery of polyphenols from marine microalgae.

Microalgae	Pre-Treatment	Solvent(s)	SLE Parameters	Extraction Time × Cycles	Phenolic Content	Ref
*Amphidinium carterae*	HSH + Alkaline–acid hydrolysis	Ethanol 70%	S/L ratio (*w*/*v*): 1:10 (2 g in 20 mL). Temperature: 1) 4 °C and 2) RT	1) 12 h × 1 2) 1 h × 1	TPC = 6.45 (free phenolics)—26.03 (bound phenolics) mg GAE/g DW. See Table 4 for more information.	[17]
*Chaetoceros calcitrans*	Freeze-dried, ground using a pestle and mortar	Ethanol/water (3:1 *v*/*v*)	S/L ratio (*w*/*v*): 1:10 (0.2 g in 2 mL). Temperature: RT	30 min × 2	TPC = 1.84 mg GAE/g DW	[11]
*Chaetoceros muelleri*	Freeze-dried, crushed using a mortar and pestle	Water, hexane, ethyl acetate	S/L ratio (*w*/*v*): 1:100 to 1:1000 (0.01–0.1 g in 10 mL). Temperature: ice	Not reported	TPC = 0.08 (hexane)—0.22 (ethyl acetate) mg/g DW	[53]
*Chaetoceros* sp.	Freeze-dried	Ethanol	S/L ratio (*w*/*v*): 1:100 (1 g in 100 mL). Temperature: RT	3 h × 3	TPC = 11.9 mg GAE/g EW	[28]
	Freeze-dried	Hexane, dichloromethane, chloroform, methanol	S/L ratio (*w*/*v*): 1:250 *w*/*v* (2 g in 500 mL). Temperature: not reported.	24 h × 1	TPC ≈ 50 (methanol)—650 (hexane) µmol GAE/g EW	[55]
*Chlorella minutissima*	Freeze-dried	3D water, methanol	S/L ratio (*w*/*v*): 1:40 (0.05 g in 2 mL). Temperature: 80 °C (3D water), RT (MeOH)	20 min × 3	TPC = 2.81 (water)—9.04 (MeOH) mg GAE/g DW.	[115]
	Freeze-dried	Hexane, dichloromethane, and ethanol	S/L ratio (*w*/*v*): 1:40 (1 g in 40 mL). Temperature: not reported	48 h × 3	TPC = 4.89 (hexane)—13.35 (EtOH) mg GAE/g DW. TFC: 10.21 (hexane)—20.65 (dichloromethane) mg QE/g DW	[109]
	Freeze-dried + HSH	Methanol and hexane	S/L ratio (*w*/*v*): 1:40 (1 g in 40 mL). Temperature: RT	overnight × 1	TPC = 3.1 mg GAE/g DW	[34]
*Chlorella* sp.	Freeze-dried and milled	Methanol/water and ethanol/ water (80:20 *v*/*v* and 50:50 *v*/*v*)	S/L ratio (*w*/*v*): 1:20 (0.075 g in 1.5 mL). Temperature: RT and 40 °C	30 min × 3	TPC = 5.88–5.90 mg GAE/g DW TFC = 6.16–6.52 mg CT/g DW	[33]
	Freeze-dried, ground using a pestle and mortar	Ethanol/water (3:1 *v*/*v*)	S/L ratio (*w*/*v*): 1:10 (0.2 g in 2 mL). Temperature: RT	30 min × 2	TPC = 1.47–2.21 mg GAE/g DW	[11]
	Freeze-dried	Ethanol	S/L ratio (*w*/*v*): 1:100 (1 g in 100 mL). Temperature: RT	3 h × 3	TPC = 8.1 mg GAE/g EW	[28]
	Freeze-dried and pulverized (to pass through a 0.8-mm screen)	Acetone 80% acidified (1% HCl)	S/L ratio (*w*/*v*): 1:100 (0.1 g in 10 mL). Temperature: 25 °C	6 h × 1	TPC = 7.7 mg GAE/g DW	[54]
*Coccolithophorid* sp.	HSH + Alkaline–acid hydrolysis	Ethanol 70%	S/L ratio (*w*/*v*): 1:10 (2 g in 20 mL). Temperature: 1) 4 °C and 2) RT	1) 12 h × 1 2) 1 h × 1	TPC = 3.76 (free phenolics)—19.63 (bound phenolics) mg GAE/g DW. See Table 4 for more information.	[17]
*Crypthecodinium cohnii*	Ground freeze-dried	Hexane, ethyl acetate, water	S/L ratio (*w*/*v*): 1:10 (0.2 g in 2 mL). Temperature: RT (hexane and ethyl acetate) and 80 °C (water)	30 min × 2	TPC = 2.70–12.68 (hexane), 0.85–1.12 (ethyl acetate), 0.95–2.55 (water) mg GAE/g DW.	[36]
*Dunaliella tertiolecta*	HSH + Alkaline–acid hydrolysis	Ethanol 70%	S/L ratio (*w*/*v*): 1:10 (2 g in 20 mL). Temperature: 1) 4 °C and 2) RT	1) 12 h × 1 2) 1 h × 1	TPC = 4.67 (free phenolics)—12.49 (bound phenolics) mg GAE/g DW. See Table 4 for more information.	[17]
	freeze-dried, crushed using a mortar and pestle	Water, hexane, ethyl acetate	S/L ratio (*w*/*v*): 1:100 to 1:1000 (0.01–0.1 g in 10 mL). Temperature: ice	Not reported	TPC ≈ 0.068 (hexane)—0.17 (water) mg GAE/g DW	[53]
*Dunaniella salina*	freeze-dried	3D water, methanol	S/L ratio (*w*/*v*): 1:40 (0.05 g in 2 mL). Temperature: 80 °C (3D water), RT (MeOH)	20 min × 3	TPC = 1.30 (MeOH)—8.78 (water) mg GAE/g DW.	[115]
	freeze-dried	Ethanol	S/L ratio (*w*/*v*): 1:100 (1 g in 100 mL). Temperature: RT	3 h × 3	TPC = 19.3 mg GAE/g EW	[28]
	freeze-dried, crushed using a mortar and pestle	Water, hexane, ethyl acetate	S/L ratio (*w*/*v*): 1:100 to 1:1000 (0.01–0.1 g in 10 mL). Temperature: ice	Not reported	TPC = 0.21 (water)—1.54 (ethyl acetate) mg GAE/g DW,	[53]
*Dunaniella* sp.	freeze-dried	Ethanol	S/L ratio (*w*/*v*): 1:100 (1 g in 100 mL). Temperature: RT	3 h × 3	TPC = 14.0 mg GAE/g EW	[28]
*Isochrysis galbana*	freeze-dried	3D water, methanol	S/L ratio (*w*/*v*): 1:40 (0.05 g in 2 mL). Temperature: 80 °C (3D water), RT (MeOH)	20 min × 3	TPC = 1.78 (MeOH)—8.13 (water) mg GAE/g DW	[115]
	freeze-dried, crushed using a mortar and pestle	Water, hexane, ethyl acetate	S/L ratio (*w*/*v*): 1:100 to 1:1000 (0.01–0.1 g in 10 mL). Temperature: ice	Not reported	TPC ≈ 0.10 (hexane)—0.32 (ethyl acetate) mg GAE/g DW	[53]
*Isochrysis* sp.	Freeze-dried, ground using a pestle and mortar	Ethanol/water (3:1 *v*/*v*)	S/L ratio (*w*/*v*): 1:10 (0.2 g in 2 mL). Temperature: RT	30 min × 2	TPC = 2.67–4.57 mg GAE/g DW	[11]
	Freeze-dried	Ethanol	S/L ratio (*w*/*v*): 1:100 (1 g in 100 mL). Temperature: RT	3 h × 3	TPC = 13.4 mg GAE/g EW	[28]
*Nannochloropsis gaditana*	Freeze-dried and milled	Methanol/water and ethanol/water (80:20 *v*/*v* and 50:50 *v*/*v*)	S/L ratio (*w*/*v*): 1:20 (0.075 g in 1.5 mL). Temperature: RT and 40 °C	30 min × 3	TPC = 4.92–5.13 mg GAE/g DW TFC = 5.87–6.24 mg CT/g DW	[33]
	Freeze-dried	Ethanol	S/L ratio (*w*/*v*): 1:100 (1 g in 100 mL). Temperature: RT	3 h × 3	TPC = 32.0 mg GAE/g EW	[28]
*Nannochloropsis granulata*	Freeze-dried	3D water, methanol	S/L ratio (*w*/*v*): 1:40 (0.05 g in 2 mL). Temperature: 80 °C (3D water), RT (MeOH)	20 min × 3	TPC = 1.30 (MeOH)—3.81 (water) mg GAE/g DW.	[115]
	Freeze-dried and pulverized (to pass through a 0.8-mm screen)	Acetone 80% acidified (1% HCl)	S/L ratio (*w*/*v*): 1:100 (0.1 g in 10 mL). Temperature: 25 °C	6 h × 1	TPC = 6.0–8.0 mg GAE/g DW	[54]
	Freeze-dried	Methanol	S/L ratio (*w*/*v*): 1:10 to 1:100 (1–10 g in 100 mL). Temperature: not reported.	Not reported	TPC = 43.6 μmol GAE/g EW	[116]
*Nannochloropsis oculata*	Freeze-dried	Methanol	S/L ratio (*w*/*v*): 1:100 (0.05 g in 5 mL). Temperature: RT	45 min × 2	Protocatechuic acid hexoside, quinic acid derivative, quercetin pentoside hexoside, luteolin 7-O-glucoside, chicoric acid derivative, caffeoyl hexoside deoxyhexoside	[44]
	Freeze-dried, ground using a pestle and mortar	Ethanol/water (3:1 *v*/*v*)	S/L ratio (*w*/*v*): 1:10 (0.2 g in 2 mL). Temperature: RT	30 min × 2	TPC = 2.04 mg GAE/g DW	[11]
	Freeze-dried + HSH	Methanol and hexane	S/L ratio (*w*/*v*): 1:40 (1 g in 40 mL). Temperature: RT	overnight × 1	TPC = 4.1 mg GAE/g DW	[34]
*Nannochloropsis salina*	HSH + Alkaline–acid hydrolysis	Ethanol 70%	S/L ratio (*w*/*v*): 1:10 (2 g in 20 mL). Temperature: 1) 4 °C and 2) RT	1) 12 h × 1 2) 1 h × 1	TPC = 2.78 (free phenolics)—21.32 (bound phenolics) mg GAE/g DW. See Table 4 for more information.	[17]
*Nannochloropsis* sp.	Freeze-dried, ground using a pestle and mortar	Ethanol/water (3:1 *v*/*v*)	S/L ratio (*w*/*v*): 1:10 (0.2 g in 2 mL). Temperature: RT	30 min × 2	TPC = 1.39 mg GAE/g DW	[11]
	Freeze-dried	Hexane, dichloromethane, chloroform, methanol	S/L ratio (*w*/*v*): 1:250 *w*/*v* (2 g in 500 mL). Temperature: not reported.	24 h × 1	TPC ≈ 50 (chloroform)—160 (hexane) µmol GAE/g EW	[55]
	PEF—1% suspension	DMSO (30%, 50%, and 100%), ethanol (30%, 50%, and 100%)	S/L ratio (*w*/*v*): 1:100. Temperature: RT	240 min × 1	Not reported	[48]
	Freeze-dried, crushed using a mortar and pestle	Water, hexane, ethyl acetate	S/L ratio (*w*/*v*): 1:100 to 1:1000 (0.01–0.1 g in 10 mL). Temperature: ice	Not reported	TPC ≈ 0.08 (water)—0.6 (ethyl acetate) mg GAE/g DW	[53]
*Navicula* sp.	Freeze-dried	Ethanol	S/L ratio (*w*/*v*): 1:100 (1 g in 100 mL). Temperature: RT	3 h × 3	TPC = 19.7 mg GAE/g EW	[28]
	HSH + Alkaline–acid hydrolysis	Ethanol 70%	S/L ratio (*w*/*v*): 1:10 (2 g in 20 mL). Temperature: 1) 4 °C and 2) RT	1) 12 h × 1 2) 1 h × 1	TPC = 2.60 (free phenolics)—5.16 (bound phenolics) mg GAE/g DW. See Table 4 for more information.	[17]
*Nitzschia laevis*	Ground freeze-dried	Hexane, ethyl acetate, water	S/L ratio (*w*/*v*): 1:10 (0.2 g in 2 mL). Temperature: RT (hexane and ethyl acetate) and 80 °C (water)	30 min × 2	TPC = 2.37 (hexane, ethyl acetate)—3.88 (water) mg GAE/g DW.	[36]
*Pavlova lutheri*	Freeze-dried, crushed using a mortar and pestle	Water, hexane, ethyl acetate	S/L ratio (*w*/*v*): 1:100 to 1:1000 (0.01–0.1 g in 10 mL). Temperature: ice	Not reported	TPC ≈ 0.1 (hexane)—0.25 (ethyl acetate) mg GAE/g DW	[53]
*Pavlova salina*	Freeze-dried, crushed using a mortar and pestle	Water, hexane, ethyl acetate	S/L ratio (*w*/*v*): 1:100 to 1:1000 (0.01–0.1 g in 10 mL). Temperature: ice	Not reported	TPC ≈ 0.07 (hexane)—0.32 (ethyl acetate) mg GAE/g DW	[53]
*Phaeodactylum tricornutum*	Freeze-dried, ground using a pestle and mortar	Ethanol/water (3:1 *v*/*v*)	S/L ratio (*w*/*v*): 1:10 (0.2 g in 2 mL). Temperature: RT	30 min × 2	TPC = 3.19–3.75 mg GAE/g DW	[11]
	Freeze-dried	Ethanol	S/L ratio (*w*/*v*): 1:100 (1 g in 100 mL). Temperature: RT	3 h × 3	TPC = 16.8 mg GAE/g EW	[28]
	Freeze-dried and pulverized (to pass through a 0.8-mm screen)	Acetone 80% acidified (1% HCl)	S/L ratio (*w*/*v*): 1:100 (0.1 g in 10 mL). Temperature: 25 °C	6 h × 1	TPC = 9.9 mg GAE/g DW	[54]
	PEF—1% suspension	Water and DMSO 50%	S/L ratio (*w*/*v*): 1:200. Temperature: RT	4 h × 1 and 24 h × 1	TPC ≈ 8 mg GAE/g DW	[70]
	Freeze-dried	Methanol	S/L ratio (*w*/*v*): 1:10 to 1:100 (1–10 g in 100 mL). Temperature: not reported.	Not reported	TPC = 44.7 μmol GAE/g EW	[116]
*Porphyridium cruentum*	Freeze-dried, ground using a pestle and mortar	Ethanol/water (3:1 *v*/*v*)	S/L ratio (*w*/*v*): 1:10 (0.2 g in 2 mL). Temperature: RT	31 min × 2	TPC = 1.22 mg GAE/g DW	[11]
*Proteomonas sulcata*	HSH + Alkaline–acid hydrolysis	Ethanol 70%	S/L ratio (*w*/*v*): 1:10 (2 g in 20 mL). Temperature: 1) 4 °C and 2) RT	1) 12 h × 1 2) 1 h × 1	TPC 1.83 (free phenolics)—4.03 (bound phenolics) mg GAE/g DW. See Table 4 for more information.	[17]
*Schizochytrium mangrovei*	Ground freeze-dried	Hexane, ethyl acetate, water	S/L ratio (*w*/*v*): 1:10 (0.2 g in 2 mL). Temperature: RT (hexane and ethyl acetate) and 80 °C (water)	30 min × 2	TPC = 0.01 (ethyl acetate)—2.22 (hexane) mg GAE/g DW	[36]
*Schizochytrium* sp.	Ground freeze-dried	Hexane, ethyl acetate, water	S/L ratio (*w*/*v*): 1:10 (0.2 g in 2 mL). Temperature: RT (hexane and ethyl acetate) and 80 °C (water)	30 min × 2	TPC = 13.61 (hexane)—0.96 (ethyl acetate) mg GAE/g DW	[36]
	Freeze-dried, ground using a pestle and mortar	Ethanol/water (3:1 *v*/*v*)	S/L ratio (*w*/*v*): 1:10 (0.2 g in 2 mL). Temperature: RT	30 min × 2	TPC = 1.94 mg GAE/g DW	[11]
*Stauroneis* sp. *LACW24* and *Phaeothamnion* sp. *LACW34*.	Pellets suspended in 2 mL solvent + Bead beater (45 s, full power)	Ethanol 50%	S/L ratio (*w*/*v*): not reported. Temperature: RT	20 min × 2	*Stauroneis* sp. ≈ 3 mg GAE/L *Phaeothamnion* sp. ≈ 0.6 mg GAE/L	[69]
*Synechococcus* sp.	Ground freeze-dried	Hexane, ethyl acetate, water	S/L ratio (*w*/*v*): 1:10 (0.2 g in 2 mL). Temperature: RT (hexane and ethyl acetate) and 80 °C (water)	30 min × 2	TPC = 2.12 (hexane) 5.64 (ethyl acetate) mg GAE/g DW	[36]
*Tetraselmis chuii*	Freeze-dried and pulverized (to pass through a 0.8-mm screen)	Acetone 80% acidified (1% HCl)	S/L ratio (*w*/*v*): 1:100 (0.1 g in 10 mL). Temperature: 25 °C	6 h × 1	TPC = 20 mg GAE/g DW	[54]
	PEF—1% suspension	Water and DMSO 50%	S/L ratio (*w*/*v*): 1:200. Temperature: RT	4 h × 1 and 24 h × 1	TPC = 4.38–6.70 mg GAE/g DW	[70]
	Freeze-dried + HSH	Methanol and hexane	S/L ratio (*w*/*v*): 1:40 (1 g in 40 mL). Temperature: RT	overnight × 1	TPC = 8.6 mg GAE/g DW	[34]
	Freeze-dried	Methanol	S/L ratio (*w*/*v*): 1:10 to 1:100 (1–10 g in 100 mL). Temperature: not reported.	Not reported	TPC = 57.5 μmol GAE/g EW	[116]
	Freeze-dried, crushed using a mortar and pestle	Water, hexane, ethyl acetate	S/L ratio (*w*/*v*): 1:100 to 1:1000 (0.01–0.1 g in 10 mL). Temperature: ice	Not reported	TPC ≈ 0.12 (water)—0.56 (ethyl acetate) mg GAE/g DW	[53]
*Tetraselmis* sp.	Freeze-dried, ground using a pestle and mortar	Ethanol/water (3:1 *v*/*v*)	S/L ratio (*w*/*v*): 1:10 (0.2 g in 2 mL). Temperature: RT	30 min × 2	TPC = 3.74 mg GAE/g DW	[11]
	Freeze-dried	Ethanol	S/L ratio (*w*/*v*): 1:100 (1 g in 100 mL). Temperature: RT	3 h × 3	TPC = 25.5 mg GAE/g EW	[28]
	Freeze-dried, crushed using a mortar and pestle	Water, hexane, ethyl acetate	S/L ratio (*w*/*v*): 1:100 to 1:1000 (0.01–0.1 g in 10 mL). Temperature: ice	Not reported	TPC ≈ 0.12 (water)—0.50 (ethyl acetate) mg GAE/g DW	[53]
*Tetraselmis suecica*	Freeze-dried, ground using a pestle and mortar	Ethanol/water (3:1 *v*/*v*)	S/L ratio (*w*/*v*): 1:10 (0.2 g in 2 mL). Temperature: RT	30 min × 2	TPC = 3.75 mg GAE/g DW	[11]
	HSH + Alkaline–acid hydrolysis	Ethanol 70%	S/L ratio (*w*/*v*): 1:10 (2 g in 20 mL). Temperature: 1) 4 °C and 2) RT	1) 12 h × 1 2) 1 h × 1	TPC = 4.72 (free phenolics)—11.67 (bound phenolics) mg GAE/g DW. See Table 4 for more information.	[17]
	Freeze-dried, crushed using a mortar and pestle	Water, hexane, ethyl acetate	S/L ratio (*w*/*v*): 1:100 to 1:1000 (0.01–0.1 g in 10 mL). Temperature: ice	Not reported	TPC ≈ 0.205 (water)—0.77 (ethyl acetate) mg GAE/g DW	[53]
*Thraustochytrium* sp.	Ground freeze-dried	Hexane, ethyl acetate, water	S/L ratio (*w*/*v*): 1:10 (0.2 g in 2 mL). Temperature: RT (hexane and ethyl acetate) and 80 °C (water)	30 min × 2	TPC = 1.22 (ethyl acetate)—4.00 (hexane) 1.22 (ethyl acetate) mg GAE/g DW	[36]
*Tisochrysis lutea*	Freeze-dried	3D water, methanol	S/L ratio (*w*/*v*): 1:40 (0.05 g in 2 mL). Temperature: 80 °C (3D water), RT (MeOH)	20 min × 3	TPC = 1.25 (MeOH)—4.75 (water) mg GAE/g DW	[115]

#### 3.3.1. Ultrasound-Assisted Extraction (UAE)

Ultrasound-Assisted Extraction (UAE) applies ultrasound technology with suitable solvents to extract bioactive compounds [117]. The mechanical effect of the acoustic cavitation phenomena on cell disruption, as explained in Section 2.2.1, enables the permeability of the cell walls and transfer of intracellular phytochemicals into the solvent [32,117], leading to increased extraction yields than those obtained by conventional methods [41,66,118]. Due to its simplicity, low cost, scale-up ability, and reduced solvent use and extraction times [32,66,118,119], UAE is being extensively employed in current microalgae research as a “greener” alternative [120] for the extraction of polyphenols from different species, including those of marine origin [18,20,28,29,35,115]. The extraction is performed in an ultrasonic bath or with an ultrasonic probe. The bath is most frequently used since it allows the extraction of a larger number of samples simultaneously [118] and has been reported in several research works for the extraction of polyphenols from marine microalgae including *Nannochloropsis oculata* [29], *Nannochloropsis salina* [20], *Nannochloropsis gaditana* [33], *Phaeodactylum tricornutum* [18,20], *Porphyridium purpureum* [18,29], *Dunaniella salina* [20], while a probe was reported to be used on *Nannochloropsis* sp. [68].

Among the physical factors that affect the extraction efficiency of UAE, power (W), frequency (kHz) and time play a key role. The power applied influences the ultrasonic amplitude, increasing the shear forces involved, and extraction yield consequently [118]. One study on *Nannochloropsis* sp. assessed the effect of the ultrasonic power (100 W, 200 W and 400 W) on the phenolic extraction, proving that higher power (400 W) resulted in higher yields [68]. A power of 400 W has been commonly reported in studies on UAE marine-derived polyphenols [28,35,68], but lower powers such as 100 W [18] and 300 W [29] have also been employed. Nonetheless, this parameter should be carefully adjusted since higher power could degrade phenolic compounds by increased radical generation [118]. The frequency directly affects the size of exploding cavitation bubbles, mostly those between 20 to 100 kHz [118,121]. A fixed frequency of 40 kHz has been commonly employed when the extraction is performed in ultrasonic baths [18,20,29], while a lower frequency of 20–24 kHz was set when a probe was used for extraction [28,68]. Another parameter to optimize is the time of extraction to avoid degrading compounds due to prolonged exposure [118]. The mentioned study on *Nannochloropsis* sp. [68] also involved varying the extraction time from 0 to 30 min. The results showed that increasing the extraction time led to higher TPC yields until 5 min, after which a saturation point was reached. Slight increases in TPC values were observed at 15 min. While researchers did not observe any phenolic degradation phenomena related to time, similar to studies conducted on microalgae *Spirulina platensis* [66], *Scenedesmus* sp. and *Chlorella* sp. [45], other studies on different vegetal matrices have reported a decrease in the quantities of phenolic acids as extraction time increases beyond 5 min [122], the reason why studies focused on the quantification of individual phenolic compounds rather than solely on TPC values should be taken into account for future optimization advancements.

Table 6 shows the optimized conditions for the UAE of phenolic compounds from different marine microalgae with results of phenolic content found in the resulting extracts, either expressed in TPC values or as individual phenolic compounds identified and/or quantified. This comparative analysis indicates that 15 min, 400 W and 40 kHz are the most frequently used parameters for the extraction of polyphenols. Longer extraction times of 30 to 45 min have been reported, but attention must be paid to degradation since UAE is related not only to radical generation but also to an increase of the temperature throughout processing to final temperatures of 34–65 °C [66,68], so controlling and monitoring the temperature is necessary. Although this increase in temperature has been reported to have a positive effect on the extraction.

#### 3.3.2. Microwave-Assisted Extraction (MAE)

Extraction of several marine resources, such as polyunsaturated fatty acids (PUFAs) [123,124,125], phycobiliproteins [126] and carotenoids [124,127], has been successfully achieved through the application of high-frequency electromagnetic waves (2.45 GHz) [124]. The rotational movements of water and other polar compounds generated by MAE irradiation cause the dielectric heating of the sample, evaporation of water and consequential pressure-induced cell disruption and release of compounds [30]. Considered an environmentally friendly option, MAE has shown higher extractive efficiency with a reduction in time and solvent use [30,128].

Several operational factors influence the efficiency of MAE, including temperature, power, choice of solvent, solid–liquid ratio, and the type of microalgae [30,102]. Gilbert-López et al. [105] conducted an experimental design of MAE on marine microalgae *Phaeodactylum tricornutum* to assess the impact of factors such as temperature (30, 100, 170 °C), solvent composition (ethanol 0, 50, 100%), and time (2, 11, 20 min) on the recovery of phenolic compounds (expressed as TPC). Findings showed that the solvent composition and temperature were the main factors influencing extraction, while the extraction time had no significant effect. The best conditions for maximizing the yields of total phenolic compounds by MAE from *Phaeodactylum tricornutum* were determined to be 30 °C, 100% ethanol, and 2 min, obtaining a TPC value of 46.57 mg GAE/g of the extract weight (EW), as shown in Table 7. This result demonstrated the technique’s advantage in drastically reducing extraction times compared to other methods, as revealed when MAE was confronted with PLE in the same study.

Research on the application of MAE to extract phenolic compounds from marine microalgae remains limited to date. Some other studies have also reported their recovery by applying MAE on samples of other species of microalgae such as *Arthrospira platensis* [41], *Chlorella vulgaris* [49,129], *Scenedesmus obliquus* [46,130] and *Scenedesmus incrassatulus* [129].

#### 3.3.3. Pressurized Liquid Extraction (PLE)

Pressurized Liquid Extraction (PLE), also referred to as Accelerated Solvent Extraction (ASE), has been employed as a method to enhance the mass transfer and solubility of phytochemicals into solvents through the application of elevated pressure (ranging from 3.3 to 20.3 MPa) and high operational temperatures (ranging from 40 to 200 °C) [32], thus enhancing the kinetics of solubilization of the matrix [99,105,131]. Before extraction, the microalgae biomass requires a preparation step in which the sample is homogenously mixed with an inert diluent (like sand) to avoid the presence of dead spaces inside the cells, ensuring the extraction and sample purity [32,131].

To maximize the efficiency of this method, parameters such as solvent selection, temperature, time, solvent flow rate and sample packing inside the cells should be optimized [32]. Among the mentioned parameters, solvent selection plays an essential role in the extraction process [32]. Some marine microalgae biomasses have been subjected to PLE to extract polyphenols, including *Phaeodactylum tricornutum* [52,105], *Tetraselmis chuii* [107] and *Chlorella* sp. [52], in extraction times ranging from 15 to 20 min at a pressure of 100–105 bar, as shown in Table 8. Optimization of operational parameters using Response Surface Methodology (RSM) has been conducted to improve the efficiency of the extraction from these matrices. Wang et al. [52] tested the solvent efficiency of DMSO at different compositions (DMSO 0, 30, 50, and 100%) on the PLE of polyphenols from *Phaeodactylum tricornutum* and *Chlorella*. The experimental design showed that solvent percentage significantly influenced the release of polyphenols from these biomasses. Increasing the solvent concentration over 30% resulted in higher recovery rates, reaching the maximum TPC at 100% DMSO with values of 11.5 mg GAE/g DW from *Phaeodactylum tricornutum* and 10 mg GAE/g DW for *Chlorella*. A similar study was conducted by Gilbert-López et al. [105] on *Phaeodactylum tricornutum*, focusing not only on the effect of the solvent composition (ethanol 0, 50, and 100%) but also on the effect of the temperature (50, 110, and 170 °C), at a fixed extraction time of 20 min and pressure of 100 bar. The authors observed that TPC increase was highly dependent on the solvent composition rather than the temperature, with higher recovery (TPC of 42.16 mg GAE/g of extract weight, corresponding to 10.14 mg GAE/g of dried biomass) with 100% ethanol at the lower temperature of 50 °C. On the contrary, another study on *Tetraselmis chuii* [107] found temperature to exert a stronger influence on the extraction by PLE rather than the solvent composition, which ranged from 0 to 100% of GRAS solvents ethyl acetate (ETAC) in cyclopentyl methyl ether (CPME). Experimental design evidenced that the highest TPC values (644.52 mg GAE/g EW = 73.28 mg GAE/g DW) were obtained at 180 °C with a solvent composition of 50% ETAC-CPME. However, the authors recalled the potential degradation of thermolabile compounds due to high temperatures.

In comparison to other extraction techniques, PLE may offer comparable polyphenol yields to those achievable with MAE [132]. Indeed, the aforementioned study on marine microalgae *Phaeodactylum tricornutum*, which compared PLE and MAE as green alternatives for recovering polyphenols, corroborates this [107]. The study demonstrated that PLE yielded 42.16 mg GAE/g EW, while MAE yielded 46.57 mg GAE/g EW at their optimal conditions. One advantage of PLE over MAE is the ability to obtain a filtered extract at the end of the process, eliminating the need for additional steps to separate the solid biomass from the extract, as required in the MAE process [132]. Compared to SFE, PLE allows for the selection of more solvents for extraction since a critical point does not have to be reached [99].

Applying PLE as an extractive technique carries several advantages from an ecological perspective, limiting the release of solvents to the atmosphere, and ensuring reduced extraction times and solvent consumption [132] compared to conventional methods. Nonetheless, PLE instrumentation is costly [32] and involves a high power consumption limiting its scale-up [30].

#### 3.3.4. Subcritical Water Extraction

Similar to PLE, the subcritical water extraction (SWE) technique consists of the use of high pressure and temperature to extract bioactive compounds, but in this case using water as an extraction solvent. The pressure applied has to be sufficient to keep water in the liquid state at operational temperatures ranging from 100 to 374 °C. Under the SWE operational conditions, the viscosity and surface tension of water are reduced, facilitating the diffusion of the solvent into the matrix [46,133]. Additionally, the dielectric constant is reduced as well, achieving a value closer to that of ethanol, which allows the dissolution of compounds of intermediate polarity like phenols [133].

To the best of our knowledge, SWE application for polyphenols recovery from marine microalgae species has not been further explored. Studies on fresh microalgae can support the efforts to evaluate the adequacy of this technique for marine-derived polyphenols. One study focused on the development of green approaches for the recovery of bioactive compounds from *Tetradesmus obliquus* found SWE to be more effective than MAE and UAE in extracting phenolic compounds [46]. The TPC level obtained by SWE (1.111 mg GAE/mL extract) was six times higher working at a constant pressure of 30 bar and a temperature of 220 °C for 20 min. Interestingly, the same study showed the best TPC results for all techniques to be achieved after subcritical CO_2_ pre-treatment.

#### 3.3.5. Supercritical CO_2_ Extraction (SC-CO_2_)

Supercritical CO_2_ Extraction (SC-CO_2_) is an environmentally friendly extraction technique in which CO_2_ behaves as a supercritical fluid for extraction, exhibiting gas-like diffusion capacity and the solvation capacity of a liquid simultaneously [62,134], allowing higher solvent penetration into the biomass [32]. Different combinations of pressure and temperature can be used to modulate the extraction of the desired compounds [32]. However, one significant drawback of this technique for its application in the isolation of polyphenols is the low polarity inherent to CO_2_, which has rendered it more appropriate and effective as a solvent for the extraction of non-polar compounds, such as carotenoids and lipids [32,62] To overcome this limitation, the addition of small percentages of polar organic solvents, such as ethanol, can be used as co-solvents during processing [32]. As an example, one study conducted on marine microalgae *Nannopclorosis gaditana* and *Tetraselmis chuii* demonstrated that the incorporation of 5 to 20% of ethanol to CO_2_ allowed the recovery of a small content of phenolic compounds (TPC lower than 5 mg GAE/g EW) that were co-extracted among a greater quantity of carotenoids [135] when working at 100 bar, 55 °C, and a CO_2_ flow rate of 20 g/min for 2 h, as seen in Table 9. Therefore, operational parameters to optimize during SC-CO_2_ extraction include the CO_2_ flow rate, pressure, temperature, extraction time and co-solvent nature and percentage [134]. A higher recovery of phenolic content might be achieved by increasing the pressure applied to 250 bar, as reported by Georgiopoulou et al. [130] in freshwater *Scenedesmus obliquus*.

SC-CO_2_ offers numerous advantages, including the recyclability, non-toxicity, and non-flammability of CO_2_ in comparison to organic solvents [134] as well as the suitability for preserving thermostable compounds [32]. Nonetheless, the most significant disadvantage that may restrict the implementation of this technique is its relatively low efficiency for extracting polyphenols due to the low polarity of CO_2_, along with the high costs associated [46].

#### 3.3.6. Comparative Evaluation of Polyphenols Extraction Techniques in Marine Microalgae

**Figure 3:**
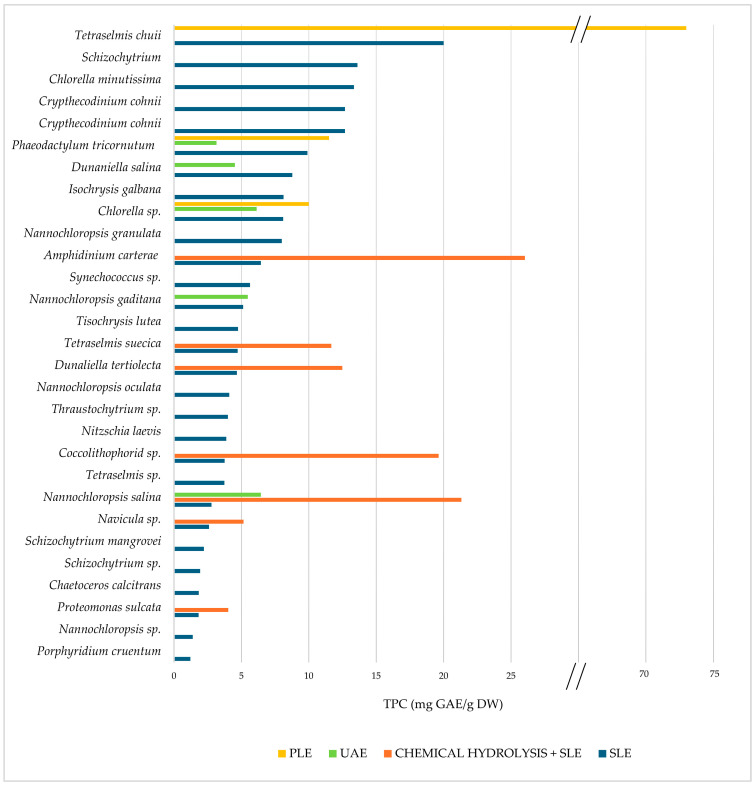
Comparative analysis of extractive techniques applied on marine microalgae for recovering phenolic compounds. The phenolic content (TPC) is expressed as mg GAE/g DW (dry-weight biomass). PLE: Pressurized-Liquid Extraction. UAE: Ultrasound-Assisted Extraction. SLE: solid–liquid extraction.

**Figure 4:**
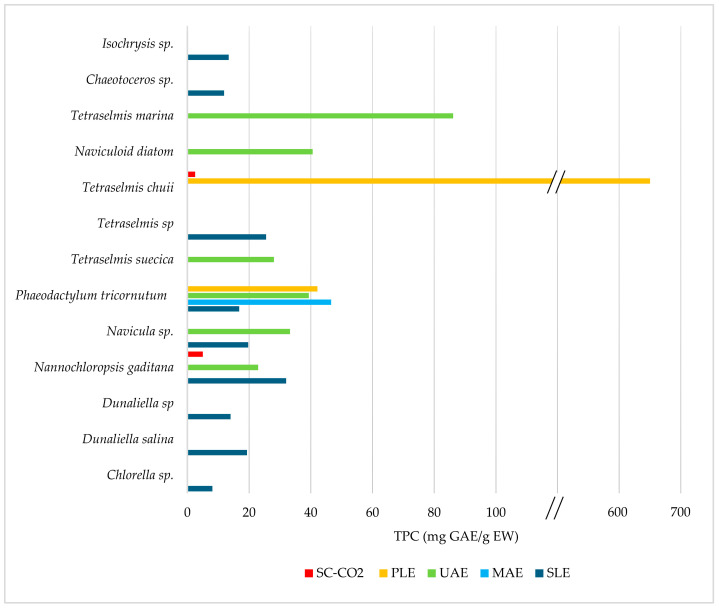
Comparative analysis of extractive techniques applied on marine microalgae for recovering phenolic compounds. The phenolic content (TPC) is expressed as mg GAE/g EW (dry extract weight). SC-CO_2_: Supercritical CO_2_ Extraction. PLE: Pressurized-Liquid Extraction. UAE: Ultrasound-Assisted Extraction. SLE: solid–liquid extraction. MAE: Microwave-Assisted Extraction.

This section presents a comparative assessment of the efficacy of different extraction techniques and treatments for the recovery of polyphenols from various marine microalgae. For this, research studies on the same type of marine microalgae mentioned in the preceding sections were collected and their polyphenol recovery was reported based on the highest TPC values found in the literature. These values were separated into two categories: those expressed as mg GAE/g DW (dry weight biomass) in Figure 3 and those expressed as mg GAE/g EW (dry extract weight) in Figure 4.

It is important to note that the differences in phenolic content observed across studies may be highly influenced by various factors, including the cultivation conditions (differences in nutrient availability, light intensity, temperature) under which the microalgae are grown, intra- and inter-species variabilities, the stage of growth (early or late stationary phase) [114,115], and the type of solvent used for extraction [28,115] as explained in Section 3.1. Nevertheless, the graphic summary presented in Figure 3 offers a comprehensive overview of the impact of diverse extraction techniques on the phenolic content concerning the biomasses obtained from various marine microalgae. The data presented suggests that chemical alkaline–acid hydrolysis treatment might be an effective approach for the recovery of higher amounts of phenolic compounds from marine microalgae, evidencing that many polyphenols in microalgae might remain bound to cellular components. Indeed, this study also confirmed the presence of individual phenolic compounds in these extracts, and thus their real extractability [17]. From unconventional extraction techniques, Pressurized Liquid Extraction (PEF) stands as an efficient novel technique for the recovery of phenolic compounds, surpassing results obtained for conventional SLE and the novel UAE, offering high extraction in short periods of 15–20 min as seen previously (Table 8). UAE efficacy to extract phenolic compounds can be in some cases comparable to SLE, which was further confirmed by Monteiro et al. [33], expressing that regardless of the technique, the solvent of extraction had more influence on the TPC recovery. However, the UAE can assist extraction by helping to recover comparable amounts of polyphenols in less time.

From this comparative analysis, it can also be seen some inter-species differences among marine microalgae. Some studies carried out on the marine microalgae *T. chuii* demonstrated that this species might be able to produce high quantities of extractable polyphenols, which was reflected in the high TPC values recovered either by SLE [54] (cultured in bioreactors) and more importantly by PLE [107] (optimal conditions achieved by response surface optimization). Figure 4 can give a better insight into the comparison of more novel techniques such as MAE and SC-CO_2_. For *Phaeodactylum tricornutum*, it was seen that MAE and PLE were as efficient in extracting polyphenols from this marine microalgae [105], and further developments can open opportunities to enhance their recovery by these methods. Due to the limitations of SC-CO_2_ regarding its low polarity, the extractability of polyphenols from marine microalgae seems to be limited. However, further studies are necessary to evaluate the real application of this technique coupled with co-solvents, pre-treatment techniques, or Response Surface Optimization studies to improve the extraction efficiency. Nonetheless, for all these techniques, phenolic identification and quantification must be necessary to confirm the presence of polyphenol subclasses in these extracts, as will be explained in the next paragraphs.

## 4. Analytical Characterization of Polyphenols

Considering the complexity of plant matrix, the low concentration of polyphenols, and their wide structural diversities resulting in different polarities and sizes (from simple phenolic acids to tannins), the analysis of these molecules is still a challenge. In fact, in addition to the complex extraction from the samples, as described in the previous sections, these characteristics of the polyphenols have significantly hindered their separation, determination, and identification. However, the identification of their chemical structure and the quantification are essential to assess their health benefits.

The next sessions summarize analytical techniques ranging from spectrophotometric techniques for semi-quantitative determination to more precise techniques for the identification and quantification of the polyphenol content in microalgae such as GC and HPLC, coupled to spectroscopic and spectrometric methods [136,137]. In particular, since the analytical characterization is performed on organic extracts that do not influence the analysis, this review reports methods that were applied mainly to marine microalgae together with a few references to freshwater microalgae.

### 4.1. Antioxidant Activity

Many methods are currently used to assess the total antioxidant activity in microalgae [138,139]. Different assay mechanisms, such as hydrogen atom transfer (HAT) and electron transfer (ET), are involved [139]. Usually, antioxidant activity is stated with assays based on chemical reactions of antioxidants with a probe, which shows a different color depending on its redox state, to get the spectrophotometric measurement.

DPPH^•^ (2,2-diphenyl-1-picrylhydrazyl) or ABTS^•+^ (2,2-azinobis-(3-ethylbenzothiazoline-6-sulfonate) radical scavenging methods are popular spectrophotometric procedures for determining the antioxidant capacities of components in microalgae extracts [140]. Other spectrophotometric methods include the ferric reducing antioxidant power (FRAP), based on the use of metal ions (Fe_3_^+^/ferricyanide complex) for oxidation, and the oxygen-radical antioxidant capacity (ORAC) assay, based on the antioxidant competition with a fluorescent probe for quenching peroxyl radicals generated from AAPH (2,2′-Azobis(2-amidinopropane) dihydrochloride) [138,141].

These spectrophotometric methods have advantages such as being simple, rapid, and precise, but they have also many disadvantages [142]. For instance, the ability of ABTS^•+^ or DPPH^•^ to react with reductants having no antioxidant activity must be considered. For instance, H_2_O_2_ can reduce DPPH^•^ to DPPHH, or, in addition, other interferences can be caused by carotenoids. Furthermore, these chemical assays measure only a fraction of the total antioxidant potential in the extract since the redox reactions have a specific redox potential. In addition, chemical methods are based on color formation that can create interferences if applied to colored chlorophylls and carotenoids algal extracts. Finally, many tests are limited for use with either lipophilic or hydrophilic extracts [143]. All these methods have their advantages and disadvantages, and one procedure cannot identify all possible mechanisms of antioxidant activities. It is essential to underline that these tests are useful to assess the total antioxidant properties of microalgal extracts, but they are not specific for the polyphenol content. When comparing the results of ET and HAT assays, often the values obtained from these measurements diverge. The differences can be attributed to many factors such as the different sensitivities of compounds to the various tests, the use of different reference standards to express antioxidant activity (gallic acid, Trolox, quercetin), and the interferences and matrix effects caused by the complex matrices [144].

Usually, to get a broad overview of the antioxidant profile, a correlation between two or more measured parameters (e.i. DPPH essay, FRAP test) with the results of the TPC is performed. This correlation can assess if phenolic compounds are a major contributor to the microalgal antioxidant capacities. It is commonly reported that high contents of phenolic compounds are associated with high antioxidant capacities [102].

Therefore, considering that these antioxidant activity tests strictly depend on the type of microalgae and must be correlated among themselves, it is not possible to make a comparison between different published works. Hence, in this paper, these antioxidant methodologies will not be further explored.

### 4.2. Spectrophotometric Determination of Phenolic Content

The Folin–Ciocalteu method, applied to measure the TPC, is a convenient, simple, reproducible, and widely used spectrophotometric assay in microalgae that has become a routine assay in studying phenolic antioxidants [138,145,146].

Due to its long history of use and acceptance in the scientific community and on the base of its reliability, the Folin–Ciocalteu assay has major popularity in comparison to other techniques. In addition, compared to other methods, the Folin–Ciocalteu assay is inexpensive, and it can be easily performed in a laboratory with common laboratory equipment. The sensitivity of the Folin–Ciocalteau assay is suitable for analyzing complex phenolic mixtures, and it allows the quantification of a wide range of phenolic compounds [144]. On the other hand, it is reported that the Folin–Ciocalteu method can interact with many substances, such as sugars, aromatic amines, sulfur dioxide, and organic acids [147] As a consequence, this test is not completely specific for polyphenols, since the Folin–Ciocalteu reagent could be reduced by other nonphenolic compounds also present in the sample, with the risk of content overestimation [142].

For example, Table 10 reports a study by Tsvetanova et al. regarding different extraction methods from the marine microalgae *Porphyridium cruentum*. Both TPC and LC-MS/MS analysis were performed on the obtained extracts. As shown by the results, the increasing TPC values did not correspond to the increasing total amount of polyphenols determined by LC-MS/MS. This may be due to the fact that the TPC results are altered by interferents [148].

Also, the total flavonoid content (TFC) is a spectrophotometric assay, widely applied for microalgae, and usually carried out colorimetrically after solvent extraction [19,60]. In general, the determination of TFC using the aluminum chloride colorimetric assay is suggested only if the absorption of the metal chelates of the individual flavonoids in a sample has a similar extinction coefficient at the maximum wavelength. In real practice, all flavonoids do not have the same absorption spectrum in the region of interest. Despite this, this test is widely used, often providing inaccurate data [149].

#### 4.2.1. Total Phenolic Content

The Folin–Ciocalteu assay is the well-known method for the determination of TPC. A mixture of tungstate and molybdate (Folin–Ciocalteu reagent), in a basic medium, oxidizes phenols. Consequently, blue-colored molybdenum ions, MoO^4+^ (λ_max_ = 750 nm) are formed. The TPC content is usually reported as gallic acid equivalent (GAE) [142,144].

In literature, the use of different procedures to remove the interferences [such as vitamin C, Cu(I), etc.] and different Folin–Ciocalteu reacting conditions to limit TPC overestimation are described [146,150,151]. Many parameters can be modified. For example, the generally used standard for calibration is gallic acid, but equivalents of catechin, tannic acid, chlorogenic acid, caffeic acid, and ferulic acid have also been employed in different matrixes such as vegetables and fruits [144,152,153]. The pH value plays a key role in the Folin–Ciocalteu assay; in fact, phenols react with the reagent only in basic conditions. For this purpose, either sodium carbonate solution, sodium hydroxide, or sodium cyanide can be successfully used. The test is time-dependent; in fact, the sample is usually incubated with the reagent for 1 h, after which the absorbance at λ_max_ (usually 760 nm) is measured for quantitative determination. Also, the incubation temperature is crucial; for instance, the blue color disappears more quickly at temperatures higher than 40 °C, even if it more rapidly appears at warmer temperatures. Usually, the solvent used in the Folin–Ciocalteu assay is water, but, for the simultaneous analysis of lipophilic and hydrophilic antioxidants, procedures using isobutanol and water mixtures with sodium hydroxide are also described [144,154,155].

In literature, the TPC of the marine microalgae is often determined by the Folin–Ciocalteu method. The applied procedures, that follow the Folin–Ciocalteu method with some modification, are almost similar even if there is a range of variation [156,157,158,159,160].

Briefly, the method consists of mixing the microalgal extract with the Folin–Ciocalteu reagent and adding a sodium carbonate solution. Then, samples are incubated. Finally, the absorbance is measured at 760 nm with a spectrophotometer. Commonly, it is reported that microalgae extracts are diluted in water, methanol, ethanol, water/ethanol and the ratio of Folin–Ciocalteau reagent with the volume of extract varies in the ratio between 1 to 8. After this addition, most papers suggest keeping the mixture at room temperature for 1 to 5 min. Then, to reach an alkaline pH, a sodium carbonate solution is added to the solution in a range of concentration from 2% to 20%, but the most common percentage is between 6% and 10%. The resulting solution is then incubated in the dark for 5 to 120 min for the reaction to occur. Usually, when the incubation time is low, a heating process (about 40–45 °C) is applied to accelerate the reaction. Then, the absorbance is measured comprising a range from 720 to 765 nm. Finally, the total phenolic content of the extracts is usually expressed in milligrams or equivalents of gallic acid (mg GAE) [11,19,28,33,36,55].

The exact chemical step and composition of the Folin–Ciocalteu reaction is still unknown, but it is supposed that the change of the initial yellow Folin–Ciocalteu reagent into blue color, is due to a series of reversible one- or two-electron reactions promoted by the phenolic compounds at basic pH. In particular, the electron-transfer reaction occurs between the phenolic compound and Mo(VI). Then, Mo^6+^ in the complex is reduced to Mo^5+^ by accepting an electron from the reducing polyphenol [144].

#### 4.2.2. Total Flavonoid Content (TFC)

After the solvent extraction from the matrix, the TFC is colorimetrically determined by adding AlCl_3_ (a complexing agent) to form Al(III)-flavonoids chelates. According to experimental conditions (such as pH value), and due to their oxo and hydroxyl groups, flavonoids demonstrate high affinity to bind metal ions [such as Al(III)]. Depending on the procedure, the absorption wavelength is set between 400 and 550 nm. In fact, NaNO_2_ is often added to produce flavonoid-nitroxyl derivatives that are peculiar for causing a bathochromic shift at about 500 nm. On the other hand, in the absence of NaNO_2_, yellow-colored Al(III)-flavonoid complexes are formed. Thus, the absorbance can be measured in a lower range (410–440 nm). Even if the use of sodium or potassium acetate in conjunction with AlCl_3_ has been reported in many procedures, in some other papers the addition of these salts does not produce any effect (or minimal) on both the λ_max_ and the relative extinction coefficient [149,161,162].

The determination of TFC is performed through the preparation of standard calibration curves. Usually, quercetin, catechin, and rutin are used as standards. This test assumes that all flavonoids absorb at the same λ_max_ and have the same extinction coefficient as the standard used in calibration. However, many flavonoids show different absorption spectra characterized by specific λ_max_ and extinction coefficient values. In addition, there might be the possibility that some polyphenols do not form the complex with Al(III), causing inaccurate results. Therefore, the application of this test for the determination of TFC in plant matrices can often be unreliable. For these reasons, its use as a standard method for the determination of TFC is very limited [149,161,162].

Reported works differ significantly in many steps of the procedure. It is reported that microalgae extracts dissolved in ethanol, methanol, butanol, acetone, or dimethyl sulfoxide, can be diluted in methanol, ethanol, or water. Then, sodium nitrite or potassium acetate, usually 1 M, is added in a very different ratio “sodium nitrite or potassium acetate/microalgal extract”, which varies from 0.06 up to 4, with a predominance of ratios of 0.3 or 0.2. Some works report a stand of 5 or 6 min at room temperature [33,139,163,164,165,166,167]. Then, an ethanolic solution at 10% aluminum chloride is added with a ratio of “aluminum chloride/microalgal extract” from 0.06 to 5, but mostly the ratio is assessed around 0.3. At this stage, the addition of 1M NaOH is sometimes described to basify the solution [33,139,163,164,165,167,168,169]. Then, samples are incubated at room temperature in a very different range of times: from 5 min to 2.5 h (even if mostly the incubation is 5 min) [33,139,163,164,165,168,169,170,171,172,173,174,175,176,177,178]. An assay performed without incubation has also been described [176]. The absorbance is measured in the range from 415 to 510 and the standard calibration curve is prepared by using different standards such as quercetin, catechin, hyperoside, and rutin [19,33,139,163,164,165,168,169,171,172,173,174,175,176,177,178].

### 4.3. Separation, Identification, and Quantification of Polyphenols

Modern high-performance chromatographic techniques coupled with instrumental analysis are the most used techniques to profile and quantify polyphenols. The two most frequently applied technologies are GC and HPLC also coupled to mass- spectrometric methods (GC-MS, HPLC-MS) [37]. GC is generally used for the separation and quantification in vegetal matrices and microalgae of volatile compounds such as fatty acid methyl esters (FAMEs) but also polyphenols. Nevertheless, because of the strong polarity of these compounds, before the analysis a pre-derivatization step is required, increasing the difficulty in sample preparation [38,39,40,179]. Today, considering the polar nature of polyphenols, HPLC is the most used analytical technique for the separation and determination of polyphenols in microalgae. This technique does not need pre-derivatization, but, on the other hand, it often requires a better matrix pre-purification step. In addition, HPLC coupled to mass spectrometric detectors allows the simultaneous analysis of all components including their derivatives and degradation products [180,181,182,183,184].

#### 4.3.1. GC Methods

Only a few studies reported the direct analysis of polyphenols in microalgae by GC analysis. In addition to polyphenols extraction, clean up, and preparation before the GC analysis, it is required to derivatize them to obtain volatile and thermostable compounds. This procedure improves the separation, selectivity, and sensitivity of the GC determination. Usually, since the process is almost instantaneous, silylated derivatives are preferred for the simplicity and the speed of the reaction, and both acids and phenols groups are derivatized in the same step. In addition, there are only a few side products that do not cause interference with the analysis. Only the use of GC-MS allows the direct characterization of flavonoids without derivatization, but it has not yet been reported for marine microalgae studies. Since the matrix is highly complex, a limitation is that peaks can sometimes be overlapped. Helium or hydrogen are commonly used as carrier gas and, in most studies, the detectors used are MS or flame ionization (FID) detectors [185]. In Table 11 papers reporting the determination of polyphenols in marine microalgae with GC are listed. In general, for the analysis of seawater and freshwater microalgae, the preferred instrumentation is GC-MS. MS parameters, when described, varied in a range of 30 degrees, and all works reported electron impact as an ionization source (EI) operating at 70 eV [186,187]. Regarding marine microalgae, different columns are used but the dimensions are similar and most of them are with these characteristics: 30 m × 0.25 mm × 0.25 μm. Helium is the most used inert transport gas at a flow of 1 mL min^−1^. The described GC oven temperature programs are different in each work. For example, Olasehinde et al. started temperature at 40 °C to arrive at 280 °C and, on the other hand, Miranda et al. started at 150 °C to reach 300 °C [40,109]. For marine microalgae, no pre-derivatization step is described in the literature. As shown in Table 11, although the analyses were directed toward the polyphenol determination in the various extracts, only very few of them were found in the analyzed samples. Miranda et al. determined phenols and polyphenols such as salicylic acid, trans-cinnamic acid, synaptic acid, chlorogenic acid, quinic acid, and caffeic acid in *Spirulina maxima* [40]. Whereas, Olasehinde et al. found no polyphenols at all [109] in *Chlorella minutissima*. Furthermore, in general, only a few papers have reported the identification of polyphenols in microalgae through GC analysis. The limited existing literature is likely due to the inefficiency of the method for determining all polyphenols in microalgal extracts, given also the complexity of the vegetal matrix. In addition, the low volatility of the molecules would require derivatization, which is often not applied, seeing the difficulties and complications in processing the samples [40,109,187].

**Table 11:** Published work regarding GC analysis of polyphenols in microalgae.

Microalgae	Instrumentation	Column	Carrier Gas/Flow	Oven/GC Parameters	Derivatization	Identified Compounds	Ref
*Spirulina maxima*	GC 500 gas chromatograph coupled to GC 300 computerized integrator, GC-FID	Capillary polymethylphenylsiloxan-FI 95 (GC do Brasil S/A, São Paulo)	Not reported	Oven: 150 °C for 3 min, increments of 5 °C min^−1^ to 300 °C	Not reported	Salicylic acid, trans-cinnamic acid, synaptic acid, chlorogenic acid, quinic acid, caffeic acid	[40]
*Chlorella minutissima*	GC–MS, Agilent 6890 series	Fused silica capillary column (30 m × 0.25 mm × 0.25 μm, coated by DB-5)	Helium/ 1 mL min^−1^	Oven: hold at 40 °C for 1 min, increase of 3 °C min^−1^ interval to 280 °C. EI operating at 70 eV Injector temperature = 250 °C.	Not reported	TPC test and FTIR analysis were positive for polyphenols, but GC-MS analysis detected many compounds, but no polyphenols	[109]

#### 4.3.2. HPLC Methods

HPLC is one of the methods of choice for the separation, identification, and quantification of polyphenols in microalgal extracts [104]. Considering the possible variations of microalgae biomass during cultivation or after processing and storage, the HPLC technique can be applied for rapid, routine, and reliable control measures. Data reporting most published methods are collected in Table 12.

Many different brands of HPLC instrumentation are reported (Varian (Palo Alto, CA, USA) Jasco (Tokyo, Japan), Hitachi (Ibaraki, Japan), Agilent (Santa Clara, CA, USA), Waters (Milford, MA, USA), and others). The apparatus can be equipped with binary, tertiary, or quaternary pumps. In addition, often the system is connected to a degasser, an autosampler, and a column oven [64,114,188,189,190]. Coupled detectors are diode array detectors (DAD) or ultraviolet detectors (UV/Vis). Furthermore, to improve the analysis, the ultra-high performance liquid chromatography (UHPLC) apparatus has been described for the polyphenols analysis in microalgae. That allows higher sensitivity, reduces solvent consumption and analysis time [29].

Usually, C18-based reverse phase (RP) columns are the most used with lengths from 50 to 250 mm, an internal diameter in the range of 2.1–4.6 mm, and particle size comprised from 1.7 to 5 µm. Considering that the extracts are often not properly purified, a precolumn can be used, usually with a similar solid matrix [20,29,44,114,190,191,192,193,194]. Few works, regarding freshwater microalgae, reported isocratic elution [111,188,195]. In fact, in marine microalgae, the separation of polyphenols is usually carried out in a gradient mode using a multi-component mobile phase consisting of an aqueous buffer and an organic solvent. The most commonly used organic solvents are methanol and acetonitrile [20,29,114,190,191,192,193,194]. To improve efficiency and resolution, the buffers are usually prepared with weak acids such as formic acid or acetic acid, to avoid ionization of phenolic compounds and the consequent peak tailing. In particular, the pH of the mobile phase must not be too low since it might lead to the hydrolysis of glycosidic polyphenols [104]. The volume of injections varies from 5 to 60 µL depending on the concentration of the solution, and the flow rate is usually in the range of 0.3–1.2 mL min^−1^. A longer column and a low flux often cause a longer elution of the peaks [191]. In particular, the reported run times are comprised in the range of 11–70 min, and these values depend also on the number of polyphenols to be separated [20,190].

The identification of polyphenols is performed through the use of analytical standards by comparing their retention times and UV spectra with those found in the sample. To quantify polyphenols it is necessary to prepare standard calibration curves [104,196,197]. For these reasons, DAD, which can acquire simultaneous UV–Vis spectra, is more reliable than UV–Vis detectors. The UV/Vis scan is usually from 190 to 600 nm [29,193] and the wavelengths are fixed depending on the maximum absorption of the compound that has to be determined. For instance, phenolic acids such as chlorogenic, caffeic, neochlorogenic, and gallic acid can be determined at 280 nm, some flavonoids such as apigenin, pinocembrin, and acacetin are at 290 nm and other flavonoids such as luteolin, quercetin, pinoquercetin, 3,3-dimethylquercetin, sakuranetin, taxifolin, methylquercetin and kaempferol can be measured at 330 nm [198]. Reported LoD and LoQ values calculated by the standard calibration curves, demonstrated a wide range of variation ranging from 0.01 µg mL^−1^ to 369.5 µg mL^−1^ for LoD and from 0.05 µg mL^−1^ to 1136.9 µg mL^−1^ for LoQ values [114,190].

Considering the huge amount and diversity of polyphenol derivatives that can be found in microalgae extracts, and the requirement of analytical standards, the HPLC methods described in the literature always reported a potential partial characterization of these compounds. Some methods reported for marine or freshwater microalgae were validated for the determination of only a few selected polyphenols, such as gallic acid, caffeic acid, (-)-epicatechin, chlorogenic acid, quercetin-3-O-rutinoside, ferulic acid and coumaric acid [29,110,111,199,200]. On the other hand, other works reported the optimization and the determination of a large variety of polyphenols. For instance, in two marine microalgal species, *Stauroneis* sp. and *Tetraselmis chuii*, Parkes et al. determined 23 polyphenols: phloroglucinol, gallic acid, protocatechuic acid, gentisic acid, 4-hydroxybenzoic acid, (+)-catechin, vanillic acid, chlorogenic acid, caffeic acid, syringic acid, (-)-epicatechin, coumaric acid, benzoic acid, ferulic acid, sinapic acid, rutin, myricetin, cinnamic acid, ellagic acid, quercetin, kaempferol, apigenin, curcumin [190].

The presence of the same type of polyphenols in published works, along with the scarcity of reports on other types, can be attributed to several factors. For instance, HPLC analysis is often performed routinely to check the variation of polyphenol content between one crop and another, to compare different species, or to test different extractive methods. In these cases, the determination of the content of one or two molecules is considered satisfactory [29,199]. Furthermore, to perform HPLC-DAD analysis it is necessary to produce calibration curves with pure standards, which may not be easily available and often very expensive. On the other hand, HPLC methods are relatively simple to apply, fast and the instrumentation is not expensive. For these reasons, it is still an analytical method of choice.

**Table 12:** Published work regarding HPLC methods for the analysis of polyphenols in microalgae.

Microalgae	Instrumentation	Detector	Column	Mobile Phase	HPLC Parameters	Run Time	Analyzed Polyphenols	LoD and LoQ	Ref
*Stauroneis* sp., *Tetraselmis chuii*	Varian ProStar HPLC binary system	ProStar 310-UV and 335-DAD	Column Phenomenex Onyx C18 (100 × 4.6 mm), guard cartridge Phenomenex Onyx C18 (10 × 4.6 mm)	A: 0.1% formic acid in deionized water B: 100% methanol.	Injection volume: 20 µL. Elution: 5% mobile phase B initial isocratic conditions for 1 min, a linear gradient to 30% mobile phase B for 3 min followed by a hold at 30% mobile phase B for 1 min, a linear gradient to 95% B mobile phase for 6 min with a hold for 30 s and a final re-equilibration step to the initial conditions for 1 min. Wavelengths: 270 nm, 373 nm.	11 min	Phloroglucinol, gallic acid, protocatheuic acid, gentisic acid, 4-hydroybenzoic acid, (+)-catechin, vanillic acid, chlorogenic acid, caffeic acid, syringic acid, (−)-epicatechin, coumaric acid, benzoic acid, ferulic acid, sinapic acid, rutin, myricetin, cinnamic acid, ellagic acid, quercetin, kaempferol, apigenin, curcumin	LoD range: 0.1 to 369.5 µg·mL^−1^ LoQ range: 0.4 to 1136.9 µg·mL^−1^	[190]
*Nodularia spumigena*	Liquid Chromatography Varian system. Ternary pump, autosampler. Star software.	DAD	Reverse phase Pursuit XRs C18 (Varian): column (250 mm × 4.6 mm, 5 μm), guard column (10 mm × 4.6 mm, 5 μm)	A: water enriched with 0.1% formic acid B: methanol	Injection volume: 20 μL. Flow rate of 1.0 mL min^−1^. Gradient elution: 15% B and increased up to 40% B in 13 min; it then changed for 1 min to 40% B and a linear gradient from 40% to 30% B for 1 min. After that, it was returned to 40% B for 1 min and kept isocratic for 2 min. Finally, it was returned for 3 min to its initial condition. Wavelengths: 270 nm, 324 nm.	18 min	Gallic acid, protocatechuic acid, (−) epicatechin, chlorogenic acid, syringic acid, (+) catechin	LoD range: 0.01468 and 0.1319 μg·mL^−1^ LoQ range: 0.04893 and 0.4395 μg·mL^−1^	[114]
*Nannochloropsis oculata*	LC- 8A Shimadzu 72	Not reported	C18 column	Not reported	Flow rate: 0.5 mL min^−1^. Gradient elution: acetonitrile-water over 15 min. Wavelengths: 254 nm.	15 min	Chlorogenic acid, caffeic acid, hydroxycinamic acid derivative, quercetin pentosidehexoside, caffeoyl dihydroxy phenyl lactoyl tartaric acid, quercetin-7-O-hexoside3-o-hexoside, caffeic acid derivative, luteolin 7-O-rutinoside, chicoric acid, protocatechuic acid hexoside, quinic acid derivative, quercetin pentosidehexoside, luteolin 7-O-glucoside, chicoric acid derivative, caffeoyl hexoside deoxyhexoside	Not reported	[44]
*Tetraselmis* sp.	HPLC-DAD (Agilent 1100 Series LC system, Germany), vacuum degasser (G1322A), quaternary pump (G1311A), autosampler (G1313A), thermostated column compartment (G1316A)	DAD (G1315B)	Mediterranea Sea18 column (150 × 2.1 mm, 5 μm) (Teknokroma, Spain)	A: methanol B: 2.5% acetic acid aqueous solution	Injection volume: 20 μL. Flow rate: 0.35 mL min^−1^. Gradient elution: 0–5 min: 10% A, 5–10 min: 10–30% A, 10–40 min: 30–90% A, 40–45 min: 90% A, 45–55 min: 90–10% A, and 55–60 min: 10% A. Wavelengths: 210, 280, 320, 350 nm.	60 min	Gallic acid, gentisic acid, p-hydroxybenzoic acid, catechin hydrate, 4-hydroxybenzaldehyde, vanillic acid, caffeic acid, chlorogenic acid, epigallocatechin gallate, syringic acid, epicatechin, p-coumaric acid, ferulic acid, salicylic acid, naringenin-7-glucoside, luteolin-7-O-glucoside, rutin, rosmarinic acid, ellagic acid, quercetin, flavone	Not reported	[194]
*Nannochloropsis* sp., *Tetraselmis chuii*, *Chaetoceros muelleri*, *Thalassiosira weissflogii*, *Tisochrysis lutea*	Agilent 1260 Infinity II liquid chromatograph (Agilent Technologies, Inc., Santa Clara, CA, USA), autosampler (G7129A), pump (G7111A)	DAD (G7115A)	SB-C18 column (50 mm × 4.6 mm with 1.8 µm, Agilent). Column temperature: 25 °C.	A: water and acetic acid, 98:2 by volume B: methanol and acetic acid, 98:2 by volume	Injection volume: 5 µL. Flow rate: 0.75 mL min^−1^. Gradient elution: 0 min at 2% B, 22 min at 40% B, 26 min maintained at 40% B, 28 min at 100% B, then at 36 min returning to 2% B. Wavelengths: from 190 to 400 nm.	36 min	Kaempferol, vitexin, rutin, p-coumaric acid, catechin, chlorogenic acid, gallic acid, p-hydroxybenzoic acid	Not reported	[193]
*Spirulina maxima*	Dionex Summit IV HPLC system, Dionex P680 dual gradient pump, ASI-100 auto-sampler	DAD-100 detector	Reversed-phase column C18 (250 × 4.6 mm, 5 μm). Column temperature: 25 °C	A: methanol/ ammonium acetate 0.1 N; 7:3, *v*/*v* B: methanol	Injection volume: 20 μL. Flow rate: 0.9 mL min^−1^. Gradient elution: 25% B, changing at 50% in 1 min, rising up to 100% B at 10 min. Wavelengths: not reported.	35 min	Gallic acid, p-hydroxybenzoic acid, chlorogenic acid, vanillic acid, caffeic acid, syringic acid, salicylic acid, o-coumaric acid, ferulic acid, cinnamic acid, quercetin, genistein, kaempferol, eugenol, chrysin, galangin, pinostrobin	Not reported	[192]
*Tetraselmis* sp.	HPLC Agilent 1260 liquid chromatograph (Agilent Technologies, Santa Clara, CA, USA), quaternary pump, autosampler	DAD (model not specified)	Symmetry C18 column (250 × 4.6 mm, 5 μm) Agilent Zorbax SB-C18, Santa Clara, USA. Column temperature: 25 °C.	A: 99.9% *v*/*v*, acetonitrile B: 0.1% *v*/*v*, acetic acid	Injection volume: 20 µL. Flow rate: 0.3 mL min^−1^. Gradient elution: 0 min, 0% A/100% B; 5 min, 15% A/85% B; 50 min, 50% A/50% B, 60 min, 100% A/0% B, 70 min, 0% A/100% B. Wavelengths: scan for 240–340 nm.	70 min	Gallic acid, epigallocatechin gallate, chlorogenic acid, catechin, caffeic acid, vanillic acid, syringic acid, coumaric acid, vanillin, sinapic acid, ferulic acid, benzoic acid, quercetin, eugenol	Not reported	[191]
*Nannochloropsis salina*, *Phaeodactylum tricornutum*, *Dunaliella salina*	Agilent 1100 Liquid Chromatograph (Agilent Technologies, Santa Clara, CA, USA)	DAD (model not specified)	Prodigy ODS-3 column (250 mm, 4.6 mm, 5 µm) Phenomenex (Torrance, CA, USA).	A: phosphoric acid in de-ionized water, pH = 3 B: acetonitrile	Injection volume: 20 µL. Flow rate: 0.9 mL min^−1^. Gradient elution: 5% of B and after 2 min increased to 40% in 20 min and again increased to 100% B at 15 min, then constant for 25 min. Wavelength: 280 nm.	70 min	Gallic acid, 2,5-dihydroxy benzoic acid, 3,4-dihydroxy benzoic acid, chlorogenic acid, catechin hydrate, genistein, p-hydroxybenzoic acid, caffeic acid, syringic acid, p-coumaric acid, ferulic acid, O-salicylic acid, cinnamic acid	Not reported	[20]
*Nannochloropsis oculata*, *Porphyridium purpureum*	Acquity UPLC system (Waters Corp., Milford; MA, USA)	DAD (model not specified)	Acquity UPLC BEH C18 column (2.1 × 100 mm, 1.7 µm; Waters Corp., Milford, MA, USA). Column temperature: 30 °C	A: formic acid 4.5% B: acetonitrile	Gradient elution: 0–12 min, 1% to 25% B; 12–12.5 min, 100% B; 12.5–13.5 min, 1% B. Wavelengths: 280 nm, 320 nm, 360 nm, UV/Vis spectra in the range of 200 to 600 nm.	12 min	(-)-Epicatechin, chlorogenic acid, quercetin-3-O-rutinoside	Not reported	[29]

#### 4.3.3. HPLC Coupled to MS Methods

In the last few years, due to their ability to determine the molecular mass and structural information of unknown molecules, HPLC coupled to mass spectrometry (HPLC-MS) is gaining an important role in the identification and characterization of polyphenols [201,202]. In comparison to HPLC with UV–Vis or DAD detector, HPLC-MS analysis can be several hundred-fold more sensitive. That allows the identification of trace-level compounds together with the quantification of phenolic compounds in complex matrices [104]. In addition, this technique can be exploited also for the characterization and identification of new polyphenols. Mass spectrometry determination is particularly versatile due to its applicability both in targeted and untargeted analyses [136]. Many interfaces can be connected to the HPLC system to ionize analytes prior to the mass analyzer. As reported in Table 13, the most used ionization technique is electrospray ionization (ESI), and polyphenols in microalgae are commonly detected and identified using the HPLC-ESI-MS systems, considering that molecules do not decompose during ionization. Chromatographic columns, often thermostatically controlled at 30 °C, are mostly C18 reversed phase with an internal diameter of 4.6 mm, a length comprised between 75 and 150 mm, and an internal diameter in the range from 2.7 to 5 μm [19,115,203]. Mobile phases are quite similar to those described in the previous paragraph of HPLC (Section 3.3.2). The run time, from 18 to 60 min, is related to the variety of analytes to be resolved, it depends on the characteristics of the column and the gradient elution. The soft ionization that maintains the molecule ion intact, can be performed in ESI positive and/or negative mode, and the MS parameters are different in each work as reported in Table 13 even if some of these are similar. For instance, the nitrogen drying gas is usually set at 13/15 L min^−1^, the nebulizer gas pressure is usually at 50 psi and the capillary voltage is in the range of 3500–4000 V [19,203,204]. As introduced before, LoD (up to 0.01 ng mL^−1^) and LoQ (up to 0.04 ng mL^−1^) values are lower than HPLC-UV/Vis systems, reaching the nanograms on milliliter ranges. Many polyphenols are determined with this technique. For instance, Klejdus et al. investigated the content of 13 phenolic compounds (gallic acid, protocatechuic acid, p-OH-benzoic acid, 4-dihydroxy-benzaldehyde, chlorogenic acid, vanillic acid, caffeic acid, syringic acid, p-OH-benzaldehyde, p-coumaric acid, ferulic acid, sinapic acid and o-coumaric acid) in many microalgae, such as the marine *Porphyridium cruentum*, to evaluate them as a potential source of functional food additives [203]. On the other hand, Wali et al. performed an LC-MS wide phytochemical screening on the extract of the marine microalgae *Nannochloropsis oculata* determining morin as the only polyphenol among many other compounds [204].

As reported in Table 13, there are many MS analyzers such as MS/MS, Orbitrap, and quadrupole time-of-flight (Q-TOF). In comparison to these, the single quadrupole mass analyzers are cheaper, with an acceptable selectivity, but with lower resolution power [205].

Reported HPLC-ESI-MS/MS methods exploited the ESI positive and/or negative mode too. The C18 column length is in the range of 150–250 mm, the diameter is from 3 to 5 mm and the particle size is from 3 to 5 μm [113,206]. During the last decades, the UHPLC system improved the analytical methods for complex marine microalgae matrices maintaining or even increasing resolution and being able to achieve faster separations than with conventional LC. For this reason, UHPLC is often coupled to MS (UHPLC-ESI-MS/MS, UHPLC-Orbitrap, UHPLC-ESI-Q-TOF) and it is used to detect polyphenols in microalgae. This system is characterized by packed columns of particle size less than 2 μm and low flow rates, in the range of 0.3–0.5 mL min^−1^ [18,136,148]. To this purpose, Goiris et al. reported a study regarding the identification of flavonoids in the marine microalgae *Diacronema lutheri*, *Tetraselmis suecica*, and *Porphyridium purpureum* in which the ionization source was set in ESI negative mode, MS/MS operated in multiple reaction monitoring (MRM) and different collision energies were applied for each polyphenol. As a result, with a LoQ in the range of 0.2–6.4 ng·g^−1^, the presence of 19 polyphenols was investigated in a run of 23 min [18]. Other works reported LC–High-Resolution Accurate Mass analysis with the use of Orbitrap mass spectrometer [148,207]. In particular, Tsvetanova et al. described a study regarding *Porphyridium cruentum* with the use of UHPLC coupled to Orbitrap with which during 37 min of run, 30 polyphenols were determined and confirmed, when possible, with pure standards [148,208].

Then, many other authors reported the use of Q-TOF coupled to HPLC or UHPLC [17,209]. Even in these cases, the characteristics of the MS parameters are different in each work, in particular regarding the mass spectrum scan and the collision energy for fragmentation (10, 15, 30, 50 eV). It is a very performing technique, in fact, as an example, Lomakool et al. with the LC-ESI-QTOF-MS/MS equipment were able to evaluate the presence of a great number of phenolic compounds (83) including phenolic acids (12), flavonoids (44), other polyphenols (22), lignans (3), and tannins (2) in the microalgal biomass (*Coelastrum* sp.) in 90 min of run. Phenolic compounds present in the microalgal extract were tentatively identified from their mass-to-charge ratios (*m*/*z* values) and MS spectra in both negative and positive ionization modes ([M + H]^−^/[M + H]^+^) [210]. As another example, Zhou et al. determined the presence of ten polyphenols in seven marine microalgal species: *Amphidinium carterae*, *Coccolithophorid* sp., *Dunaliella tertiolecta*, *Microchloropsis salina*, *Navicula* sp., *Proteomonas sulcata*, *Tetraselmis suecica* [17].

On the other hand, Asnani et al. performed LC-ESI-Q-TOF analysis to identify polyphenols too. However, flavonoids were not determined in the analyzed samples even though the presence of these compounds was supported by the results of phytochemical and TFC screening [211]. Therefore, this confirms that HPLC-MS analyses give more accurate and reliable results than spectrophotometric methods (such as TPC and TFC).

In conclusion, the use of HPLC-ESI-MS and HPLC-ESI-MS/MS is the best option for analyzing polyphenols in several matrices. However, the opportunity of access to these technologies is still restricted for most laboratories [212]. This equipment can be useful not only for targeted quantification or untargeted polyphenols but also for the analysis of comprehensive compositional profiles and fingerprints of the microalgal matrix to be exploited for classification and positioning in the market. Thus, considering the complexity of the microalgal matrix and the concentration of polyphenols that can be very low (traces), MS/MS and, in particular, Q-TOF and Orbitrap should be the equipment of choice for this kind of analysis. Q-TOF and Orbitrap allow to clarify the composition of polyphenols by furnishing their isotopic patterns, providing excellent mass accuracy together with better peak resolution, even if, to confirm the identity of individual polyphenols authentic standards should be used too [197]. From a perspective, HPLC-MS will gradually become the technique of choice for the research of polyphenols in complex matrices such as those of marine microalgae.

**Table 13:** Published work regarding HPLC/LC coupled with MS methods for the analysis of polyphenols in microalgae.

Microalgae	Instrumentation	Detector	Column	Mobile Phase	HPLC/LC Parameters	MS Parameters	Run Time	Analyzed Polyphenols	LoD and LoQ	Ref
*Nannochloropsis oculata*	Model not reported	DAD, mass spectrometer, ESI interface	Acquity C18 (100 mm × 4.6 mm × 5 µm)	A:B methanol:2mM ammonium acetate:formic acid (65:35:0.1)	Flow rate: 0.6 mL min^−1^. Isocratic elution.	Positive ion mode ionization. Voltage: 30 V. Capillary voltage: 3.5 V. Desolvation gas: 900 L h^−1^. Desolvation temperature: 400 °C. Cone gas: 50 L h^−1^. Source temperature: 150 °C. Collision energy: 22 V.	18 min	Morin	Not reported	[204]
*Phaedactylum tricornitum*, *Nannochloropsis gaditana*, *Nannochloris* sp., *Tetraselmis suecica*	Nexera X2 liquid chromatography system (Shimadzu, Kyoto, Japan), CBM-20A controller, two LC-30AD dual-plunger parallel flow pumps, DGU-20A5 degasser	DAD, Shimadzu mass spectrometer (LC-MS-2020 Shimadzu, ESI interface	Ascentis Express RP C18 (150 mm × 4.6 mm, 2.7 µm)	A: water/acetic acid (99.85/0.15) B: acetonitrile	Flow = 1 mL min^−1^. Gradient elution: 0–5 min, 5% B, 5–15 min, 10% B, 15–30 min, 20% B, 30–60 min, 50% B, 60 min, 100% B. Wavelengths: 280, 330 nm.	Positive and negative ionization modes. Scan range: *m*/*z* 100–800. Scan speed: 2500 u s−1. ESI conditions: 0.3 s event time, 1.5 L min−1 nebulizing gas (N2) flow rate 15 L min−1 drying gas (N2) flow rate, 350 °C interface temperature, 300 °C heat block temperature, 300 °C desolvation line temperature, 1V desolvation line voltage, −4.5 kV interface voltage, and 0 V Q array desolvation line voltage.	60 min	Gallic acid, caffeic acid, rutin, catechin, coumarin, kaempferol, apigenin, quercetin	LoD range: 0.20–0.97 µg mL^−1^ LoQ range: 0.67–3.25 µg mL^−1^	[19]
*Chlorella minutissima*, *Isochrysis galbana*, *Nannochloropsis oculata*, *Tisochrysis lutea*	LC Series 1260, Agilent Technologies Inc. (Santa Clara, CA, USA)	Agilent LC 1260 Infinity II DAD detector, Single Quadrupole mass analyser	POROSHELL 120 EC-C18 (4.6 mm × 150 mm, 2.7 μm). Temperature: 30 °C.	A: 0.2% formic acid in 3D H_2_O B: 0.2% formic acid in acetonitrile	Injection volume: 10 μL. Flow rate: 0.5 mL min^−1^. Gradient elution: 5 min 5% B, increase to 43% B in 10 min, for 5 min, increased to 50% B in 5 min. B in the elution solution gradually increased to 100% in 7 min, for 5 min, decreased to 5% in 2 min then ran isocratically.	Not reported	45 min	Gallic acid, protocatechuic acid, 4-hydroxybenzoic acid, 4-hydroxybenzaldehyde, vanillic acid, (+)-catechin, caffeic acid, (-)-epicatechin, p-coumaric acid, (-)-epigallocatechin 3-gallate, salicylic acid, (-)-epicatechin 3-gallate, resveratrol	Not reported	[115]
*Porphyridium purpureum*, *Diacronema lutheri*, *Tetraselmis suecica*, *Phaedactylum tricornitum*	Acquity H-class UHPLC system	MS/MS, Xevo TQ-S mass spectrometer (Waters)	Waters Acquity ethylene bridged hybrid [BEH]-shield RP18 (3.0 × 150 mm, 1.7 μm), BEH phenyl (2.1 × 100 mm, 1.7 μm). Temperature: 40 °C	A: water + 0.1% formic acid B: acetonitrile + 0.1% formic acid	Flow rate: 0.500 mL min^−1^. Gradient elution: 0% to 26% B in 9.9 min, to 65% B at 18.5 min, and to 100% B at 18.8 min and held at 100% B to 20.8 min. Re-equilibrated to initial conditions of 100% A e from 20.9 to 23 min.	Negative ESI mode. MS/MS in multiple reaction monitoring. Cone voltage: 40 V. Capillary potential: 2.8 kV and a source offset of 30 V. Source temperature: 150 °C. Desolvation temperature: 450 °C. Gas flows: 800 mL h^−1^, 150 L h^−1^ and 0.25 L h^−1^ for desolvation, cone and collision gas, respectively.	23 min	Phloroglucinol, p-coumaric acid, ferulic acid, caffeic acid, phloretin, naringenin, eriodictyol, apigenin, luteolin, daidzein, genistein, dihydrokaempferol, dihydroquercetin, kaempferol, quercetin, catechin, epicatechin, procyanidin A2, procyanidin B2	LoQ = range 0.2–6.4 ng·g^−1^	[18]
*Porphyridium cruentum*	LC–High-Resolution, TurboFlow^®^ Ultra High-Performance Liquid Chromatography (UHPLC) system (Thermo Scientific Co., Waltham, MA, USA), HTC PAL^®^ autosampler (CTC Analytics, Zwingen, Switzerland)	Accurate Mass analysis (LC–HRAM), Q Exactive^®^ hybrid quadrupole-Orbitrap^®^ mass spectrometer (Thermo Scientific Co., Waltham, MA, USA), HESI^®^ (heated electrospray ionization) module	Nucleo shell C18 (100 × 2.1 mm, 1.8 µm).	A: 0.1% formic acid in water B: 0.1% formic acid in acetonitrile	Flow rate: 300 µL min^−1^. Gradient elution: 0% B, hold for 2 min; 0–40% B—26 min, 40–90% B—3 min; 90% B—1 min; 90–0% B for 2 min and 0% B for 3 min.	Ion fragmentation in negative mode. Reaction monitoring mode. Resolution settings of 17,500 and 0.5 amu isolation window of precursor ions for quantitative analysis. Full-scan mass spectra, *m*/*z* range 100–1200, resolution settings of 70,000. Capillary temperature: 320 °C. Probe heater temperature: 300 °C. Auxiliary gas flow: 12 units. Sweep gas: 2 units. S-Lens RF level: 50.00.	37 min	o-coumaric acid, p-coumaric acid, m-coumaric acid, ferulic acid, cinnamic acid, 3-O-caffeoylquinic, chlorogenic acid, gallic acid, vanillic acid, ellagic acid, gentisic acid, protocatechuic acid, o-hydroxybenzoic acid, m-hydroxybenzoic acid, syringic acid, 3-hydroxy-4-methoxybenzoic acid, quercetin, myricitrin, myricetin, rutin, resveratrol, kaempferol, kaempferol-3-O-glycoside, fisetin, luteolin, apigenin, catechin, epicatechin, hesperidin, naringenin	Not reported	[148,208]
*Phaeocystis globosa*	Vanquish UHPLC system (Thermo Fisher, Waltham, MA, USA)	Orbitrap Q-Exactive HF-X mass spectrometer, data-dependent acquisition mode	Accucore HILIC (100 × 2.1 mm, 2.6 μm)	Not reported	Flow rate: 0.3 mL min^−1^. Gradient elution: 2.0% B, 1.0 min; 2.0–50.0% B, 16.5 min; 50.0–2.0% B, 2.5 min.	Spray voltage: 3.2 kV, Sheath gas flow rate: 35 arb. Capillary temperature: 320 °C. Aux gas flow rate: 10 arb.	20 min	Luteolin, gallic acid	Not reported	[207]
*Amphidinium carterae*, *Coccolithophorid* sp., *Dunaliella tertiolecta*, *Microchloropsis salina, Navicula* sp., *Proteomonas sulcata*, *Tetraselmis suecica*	Agilent 1200 HPLC system	Agilent 6520 Accurate-Mass Q-TOF LC/MS	Synergi Hydro-RP 80 Å, (250 mm × 4.6 mm, 4 μm particle size), guard column C18 ODS (4.0 × 2.0 mm), Phenomenex	A: water/formic acid mixture (99.8: 0.2, *v*/*v*) B: acetonitrile/water/formic acid (50: 49.8: 0.2, *v*/*v*/*v*).	Injection volume: 5 μL. Flow rate: 0.8 mL min^−1^. gradient profile used was as follows: 90% A 0 min, 90–65% A 0–30 min, 65–60% A 30–35 min, 60–45% A 35–40 min, 45–25% A 40–50 min, 25–5% A 50–55 min, 50% A 55–57 min, 0–90% A 57–60 min.	Positive and negative ionization modes. Capillary voltage: 3.5V. Nozzle voltage: 500 V.	60 min	Gallic acid, 4′-O-methyl-(-)-epigallocatechin 7-O-glucuronide, chrysoeriol 7-O-glucoside, apigenin 6,8-di-C-glucoside, quercetin 3-O-arabinoside, quercetin 3′-sulfate, cyanidin 3-O-(6″-acetyl-glucoside), scopoletin, secoisolariciresinol-sesquilignan, schisandrin	Not reported	[17]
*Coelastrum* sp.	Agilent 1200 series HPLC (Agilent Technologies, Santa Clara, CA, USA)	ESI-QTOF-MS/MS: Agilent 6545 Accurate-Mass Q-TOF LC–MS, ESI interface (Agilent Technologies, Santa Clara, CA, USA)	Poroshell 120 EC-C18, LC (2.1 × 100 mm, 2.7 µm) (Agilent Technologies, Santa Clara, CA, USA) Temperature: room temperature	A: water/acetic acid (98:2, *v*/*v*) B: acetonitrile/acetic acid/water (50:0.5:49.5, *v*/*v*/*v*).	Injection volume: 6 µL. Flow rate: 0.4 mL min^−1^. Gradient elution: 10–25% B, 0–25 min, 25–35% B, 25–35 min, 35–40% B, 35–45 min, 40–55% B, 45–75 min, 55–80% B, 75–79 min, 80–90% B, 79–82 min, 90–100% B, 82–84 min, 100–10% B, 84–87 min, and isocratic 10% B, 87–90 min.	Negative and positive ionization modes. Sample temperature: 10 °C. Nitrogen gas nebulization: 45 psi. Flow rate: 5 L min^−1^ at 300 °C. Sheath gas: 11 L min^−1^ at 250 °C. Capillary voltage: 3.5 kV. Nozzle voltage: 500 V. Mass scan from *m*/*z* 50 to 1300. Collision energy (10, 15 and 30 eV) for fragmentation. Phenolic compounds identified with Agilent LC–MS Qualitative Software and Personal Compound Database and Library.	90 min	Gallic acid, gallic acid 4-O-(6-galloylglucoside), m-trigallic acid, 3,4-O-dimethylgallic acid, p-coumaric acid, dihydro-3-coumaric acid, ferulic acid, ferulic acid 4-O glucuronide, isopeonidin 3-arabinoside, 3,4-Dihydroxyphenylacetic acid, 2-hydroxyphenylacetic acid, phenylacetic acid, delphinidin 3-(6″-malonylglucoside) 5-glucoside, malvidin 3-galactoside,delphinidin 3-glucosylglucoside, delphinidin 3-(acetylglucoside), delphinidin 3-O-3″,6″-O-dimalonylglucoside, delphinidin 3-O-β-D-glucoside 5-O-(6-coumaroyl-β-D-glucoside), isopeonidin 3-glucoside, delphinidin 3-O-(6-O-malonyl-β-D-glucoside), prodelphinidin A2 3′-gallate, gallocatechin-(4alpha- > 8)-gallocatechin-(4alpha- > 8)-gallocatechin, (-)-epigallocatechin 3-(4-methyl-gallate), prodelphinidin A1, epigallocatechin-(2b- > 7,4b- > 8)-gallocatechin, 8,8′-methylenebiscatechin, epicatechin-(4beta- > 8)-gallocatechin, 8-C-ascorbyl epigallocatechin 3-O-gallate, gallocatechin-(4alpha- > 8)-epigallocatechin, 7-galloylcatechinent-epicatechin-(4alpha- > 8)-ent-epicatechin 3,3′-digallate, 3′-galloylprodelphinidin B2, epigallocatechin-(4beta- > 8)-epicatechin-3-O-gallate ester, 8,8′-methylenebiscatechin, yetramethylquercetin 3-rutinoside, quercetin 3,7,4′-O-triglucoside, quercetin 3-(4″-acetylrhamnoside) 7-rhamnoside, quercetin 3,7-dimethyl ether, quercetin 3-(2-caffeoylsophoroside) 7-glucoside, quercetin 3,7,4′-tri-O-sulfate, quercetin 3-(6″-sinapoylsophorotrioside), quercetin, quercetin 3-galactoside, quercetin 3-O-(6-O-malonyl-β-D-glucoside), quercetin 3-O-(6″-malonylglucoside)7-O-glucoside, quercetin 3-(2-glucosylrhamnoside), quercetin 3-arabinoside, quercetin 7-glucuronide 3-rhamnoside, quercetin 3-sophoroside, quercetin 3-(2-galloylglucoside), quercetin 3-O-(6″-acetylglucoside), quercetin 3-(2-caffeoylglucuronoside), 4′-O-methyldelphinidin 3-O-beta-D-glucoside, 4′-O-methyldelphinidin 3-O-rutinoside, dalbergin, dihydrobiochanin A, 2′-hydroxyenterolactone, arctigenin, 8–8′-dehydrodiferulic acid, 3-methylellagic acid 2-(4-galactosylglucoside), guibourtinidol-(4alpha- > 6)-catechin, 5-(3′,4′-dihydroxyphenyl)-gamma-valerolactone, 4-hydroxybenzaldehyde, 2,5-dihydroxybenzaldehyde, 3-dimethylallyl-4-hydroxybenzaldehyde, isoscopoletin, dihydrocaffeic acid 3-O-glucuronide, 2-hydroxyphenylacetic acid O-b-D-glucoside, urolithin A 3, 8-O-diglucuronide, urolithin A-3-O-glucuronide, 2-hydroxybenzaldehyde O-[xylosyl-(1- > 6)-glucoside], 5-(3′,5′-dihydroxyphenyl)-gamma-valerolactone 3-O-glucuronide, carnosol, carnosic acid, 7-methylrosmanol, 11,12-dimethylrosmanol, 6,7-dimethoxy-7-epirosmanol, epirosmanol, hydroxytyrosol 1-O-glucoside, dihydrocaffeic acid 3-sulfate, phloroglucinol, dihydrophloroglucinol, leucodelphinidin 3-[galactosyl-(1- > 4)-glucoside]	Not reported	[210]

#### 4.3.4. Comparison of Marine Microalgae Analysis Methods for Polyphenols Determination

**Figure 5:**
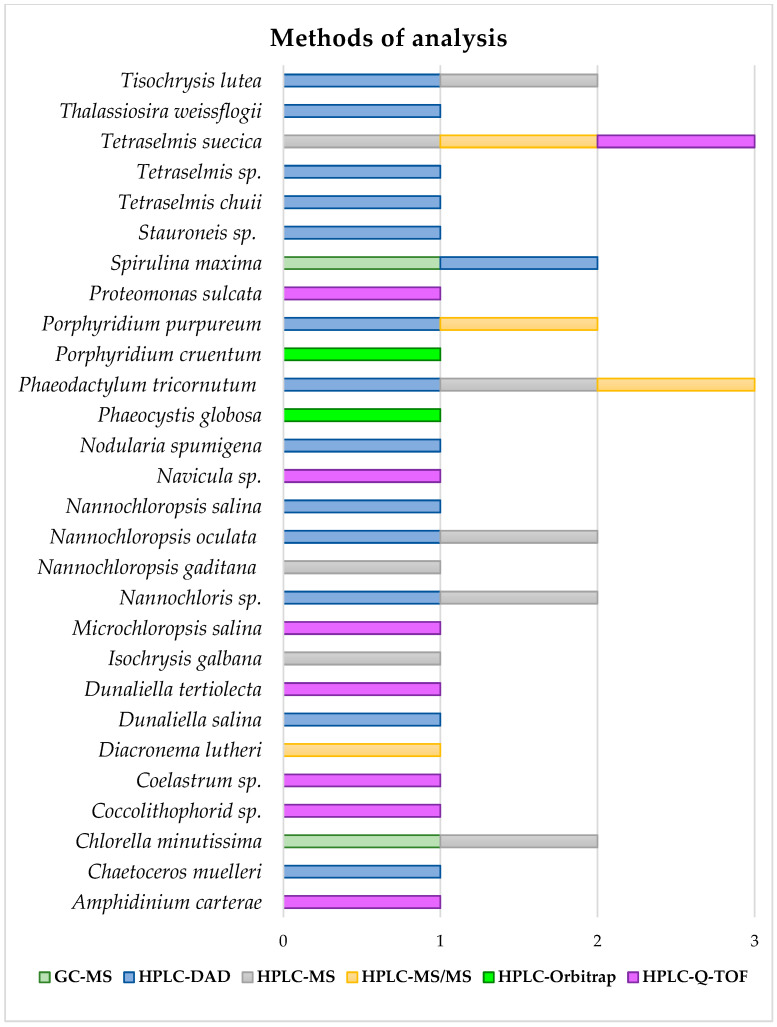
Published analytical methods regarding the research of polyphenols in marine microalgae. Microalgae are reported in alphabetic order and analytical methods are represented by different colored bars [17,18,19,20,29,40,44,115,192,193,204].

As discussed in the previous paragraphs (Section 4.3.1, Section 4.3.2 and Section 4.3.3), in the literature, not many works have reported the determination of polyphenols in marine microalgae samples. Moreover, the analyses described are incomplete, some details of the analytical method parameters are missing with partially described chromatographic conditions and the analyses concern only some polyphenols. This is probably due to the complexity of the microalgal matrix, the difficulties of analysis, the advanced instrumentation required, and the need for highly trained staff. Figure 5 represents a report of published analytical methods regarding the research of polyphenols in marine microalgae [17,18,19,20,29,40,44,115,192,193,204]. As highlighted by the colored bars, most of the analyses were conducted with HPLC equipment, and this is probably the most used analytical method due to its low cost, ease of analysis, and applicability in terms of quality control of the products. This is followed by methods obtained with HPLC-MS, HPLC-Q-TOF, HPLC-MS/MS, and only a few with HPLC-Orbitrap. These allow more accurate analyses, even untargeted, but they are increasingly more expensive instruments and need highly specialized technicians to conduct the analyses. To date, GC-MS methods are still rarely used, considering that the analyses are not so sensitive and efficient, especially if the polyphenols are not previously derivatized.

Concerning microalgae, only a few saltwater species have been analyzed for the determination of polyphenol content, and above all only one method of analysis is usually applied. Three different methods of analysis (MS, MS/MS, Q-TOF and HPLC, MS, MS/MS respectively) were applied only for *Tetraselmis suecica* and *Phaeodactylum tricornutum*. However, it would be useful to compare the analyses obtained from different equipment and methods to make a direct comparison with the polyphenols identified and their amount.

To this regard, Table 14 reports the identified polyphenols in the marine microalgae species studied in literature along with the used analytical methods [17,18,19,20,29,40,44,115,192,193,204].

More than 40 molecules of interest were identified in the different microalgal samples and even more if the derivatives are considered. However, as highlighted by the colored bars, often different polyphenols were obtained by using various methods, despite the analyzed microalgal species being the same. For instance, regarding the microalgae *Nannochloropsis* sp., derivatives of hydroxybenzoic acid and vitexin were found by HPLC-DAD analysis, while caffeoyl-coumaroyl-quinic acid, catechin, and dimethoxyflavone, protocatechuic acid were determined by HPLC-MS. Only kaempferol was determined with the two methods [19,193]. Regarding *Phaeodactylum tricornutum*, the variety of identified polyphenols with the three different methods is reported. Specifically, by using HPLC-DAD, hydroxybenzoic acid, coumaric acid, and salicylic acid were identified; whereas caffeic acid, dimethoxyflavone, p-coumaroyl tyrosine, and protocatechuic acid were determined with MS detection; finally, apigenin, daidzein, genistein, luteolin, coumaric acid, phloroglucinol were determined by using the MS/MS approach. Among all these polyphenols, only coumaric acid derivatives were found with HPLC-DAD and UHPLC-MS/MS methods [18,19,20]. In *Spirulina maxima*, the HPLC-DAD allowed the determination of 17 polyphenols; with the GC-MS method only 6 polyphenols were identified, of which 4 corresponded to those found with HPLC (caffeic acid, chlorogenic acid, cinnamic acid, and salicylic acid) [40,192]. As another example, in *Tetraselmis suecica* numerous polyphenols were determined with UHPLC-MS/MS, of which only 2 were identified in common with the MS method (apigenin and caffeic acid derivatives) and with LC-Q-TOF-MS/MS only one additional compound was identified (cyanidin 3-O-(6″-acetyl-glucoside)) [17,18,19].

Table 15 reports marine microalgae species of Table 14 highlighting their origin and solvents for the polyphenolic extracts. As can be seen, reported microalgae are all from different origins. Some are commercial, while others come from experimental cultivations under controlled conditions. The extractive solvents are mostly methanol and ethanol.

Various factors can influence the determination of different polyphenols with different methods of analysis in marine microalgae. As a first instance, even if of the same species, marine microalgae are of different origins with different cultivation conditions (Table 15). It is well known that different growth conditions (pH, CO_2_, light intensity, nutrients, stress) can stimulate the accumulation of bioactive molecules favoring some categories over others [4,213]. In addition, although the extractive solvents are similar, the extractive methods can be different. Furthermore, from the point of view of analytical methods, some of these have been optimized for the specific determination of some analytes, not even looking for the presence of other polyphenols, while some methods have been applied as untargeted, determining both polyphenols and other analytes present in microalgal extracts. For these reasons, to make a real comparison of the microalgal content, it would be necessary to conduct parallel analyses with different analytical methodologies on the same batch of microalgae cultivation.

## 5. Conclusions

Currently, research focused on the phenolic content in marine microalgae is increasing, revealing their potential as alternative sources for recovering these bioactive molecules. Extraction of polyphenols from these matrices might represent a challenge given the complex cell walls present in some species, the reason why cell disruption techniques are necessary, from which chemical alkaline–acid hydrolysis has shown promising results. Conventional techniques such as SLE are still widely applied, but long extraction times are often required. UAE can speed up extraction times with comparable recoveries. The novel technique of PLE stands out as a highly efficient method with high recoveries obtained by different authors. Studies on MAE are yet to be explored for marine microalgae, but also represent a promising method to extract polyphenols even in a few minutes. Comparison between extractive methods reported in the literature can be often misleading since phenolic content is highly dependent on cultivation conditions, species variations, and the type of solvent used. For this reason, the optimization of parameters for the selected methods is crucial, from which Response Surface Methodologies have shown to be a useful tool.

In addition to the complex extraction from samples, the complexity of some marine microalgae matrices, the low concentration, and their wide structural diversities, the analysis of polyphenols is still a challenge. The most reported analytical techniques for the identification and quantification of the polyphenol content in microalgae include spectrophotometric techniques that are suitable for semi-quantitative determinations [136,137]. These methods, such as the determination of antioxidant activities with ABTS^•+^ or DPPH^•^, are simple, rapid, and precise [142], but they may give inaccurate analysis results, by measuring only a fraction of the total antioxidant potential and interfering with other molecules present in the microalgal extracts. In addition, these tests are useful to assess the total antioxidant properties of microalgal extracts, but they are not specific for the polyphenol content. Considering that high contents of phenolic compounds are associated with high antioxidant capacities, to get a more complete overview of the antioxidant profile, usually a correlation is performed between two or more measured parameters (e.i. DHPP essay, FRAP test) and the results of the TPC [146]. To this regard, the Folin–Ciocalteu, suitable but not completely specific for analyzing phenolic mixtures, is a widely inexpensive used assay even if it may be affected by interactions with sugars, aromatic amines, sulfur dioxide, and organic acids [147]. Similarly, the TFC is a widely applied assay for microalgae, but it often provides inaccurate data since flavonoids do not all have the same absorption spectrum in the region of interest.

More precise techniques to profile and quantify polyphenols are GC and HPLC also coupled to spectrometric methods [37,137]. However, among these, GC techniques are less applied since the sample preparation process could be complicated considering the strong polarity of these compounds and the necessity of sample pre-derivatization [38,39,40,179]. Today, HPLC is the most commonly used analytical technique for the separation, identification, and quantification of polyphenols in seawater microalgae. HPLC methods are relatively simple, and fast and the instrumentation is not expensive. For these reasons, HPLC is still the analytical method of choice. In addition, this technique does not need pre-derivatization, and it allows simultaneous analysis of all components. Considering the possible variations of microalgae biomass during cultivation or after processing and storage, the HPLC-DAD technique can be applied for rapid, routine, and reliable control measures. However, HPLC-DAD methods described in literature always report a partial characterization of the content of polyphenols. It is probably due to the huge amount and diversity of polyphenols that can be found in microalgae extracts, and to the requirement of analytical standards to perform the analysis.

In the last years, due to the ability to determine the molecular mass and structural information of unknown molecules, HPLC coupled to MS, MS/MS, Orbitrap, or Q-TOF, is gaining an important role in the identification and characterization of polyphenols [201,202]. In comparison to HPLC, the HPLC coupled to spectrometric devices increases the sensitivity and it allows the identification of trace-level compounds together with the quantification of phenolic compounds in complex matrices [104]. In particular, HPLC-ESI-Q-TOF and HPLC-ESI-Orbitrap can furnish the isotopic patterns of molecules and provide excellent mass accuracy together with better peak resolution, to confirm the identity of individual polyphenols [197]. In addition, by performing targeted or untargeted analyses, these techniques can be exploited for the characterization and identification of new polyphenols and also for the analysis of compositional profiles and fingerprints of the microalgal matrix with the aim of classification and positioning in the market [136]. In conclusion, the use of HPLC coupled with MS, MS/MS, Orbitrap, or Q-TOF is the best option for the analysis of polyphenols in several matrices. However, these equipment are expensive and they need highly specialized technicians to conduct the analyses [212].

In the literature not many works report the determination of polyphenols in marine microalgae samples, described analyses are not completed, and some details of the analytical method parameters are missing. By exploiting the existing literature, it is not possible to make a real comparison of the microalgal content, since described analyses of polyphenols are obtained with different equipment and different batches of microalgae.

From a future perspective, the best procedure will be the optimization of polyphenol extractions from the complex algal matrix combined with HPLC analysis with a high-resolution mass spectrometer.

## Figures and Tables

**Table 1:** Phenolic compounds identified in main marine microalgae.

Microalgae	Phenolic Acids	Flavonoids	Lignans	Ref
*Phaeodactylum tricornutum*	p-coumaric acid, ferulic acid	Daidzein, genistein		[18]
	Protocatechuic acid, caffeic acid, caffeic acid hexoside dimer, p-coumaroyl tyrosine	Dimethoxyflavone		[19]
	3,4 dihydroxy benzoic acid, p-coumaric acid, salicylic acid			[20]
*Nannochloropsis gaditana*	Protocatechuic acid, caffeic acid, caffeoyl glucoside, feruloylglucaric acid, p-coumaroyl tyrosine	Quercetin, apigenin-O-rutinoside, rhamnosyl hexosyl-methyl quercetin		[19]
*Nannochloropsis salina*		4′-O-methyl-(-)-epigallocatechin 7-O-glucuronide, Chrysoeriol 7-O-glucoside,	Schisandrin	[17]
	Gallic acid, 3,4 dihydroxy benzoic acid, ferulic acid, p-coumaric acid, salicylic acid			[20]
*Nannochloris* sp.	Protocatechuic acid, caffeoyl coumaroyl-quinic acid	Catechin, dimethoxyflavone, kaempferol		[19]
*Tetraselmis suecica*	p-coumaric acid, ferulic acid	Apigenin, daidzein		[18]
		Cyanidin 3-O-(6″-acetyl-glucoside)		[17]
	Protocatechuic acid, caffeic acid, caffeoyl glucoside, p-coumaroyl tyrosine,	Dimethoxyflavone, apigenin-O-rutinoside, rhamnosyl hexosyl-methyl-quercetin		[19]
*Porphyridium purpureum*	p-coumaric acid, ferulic acid	Apigenin, luteolin, daidzein, genistein, quercetin		[18]
*Dunaliella tertiolecta*		4′-O-methyl-(-)-epigallocatechin 7-O-glucuronide		[17]
*Dunaliella salina*	Caffeic acid, ferulic acid, p-coumaric acid			[20]
*Diacronema lutheri*	p-coumaric acid, ferulic acid,	Apigenin, quercetin, phloretin, naringenin, dihydroquercetin, dihydrokaempherol.		[18]
*Proteomonas sulcata*	Gallic acid	4′-O-methyl-(-)-epigallocatechin 7-O-glucuronide, Apigenin 6,8-di-C-glucoside, Quercetin 3′-sulfate,	Secoisolariciresinol-sesquilignan, schisandrin	[17]
*Navicula* sp.		4′-O-methyl-(-)-epigallocatechin 7-O-glucuronide, Apigenin 6,8-di-C-glucoside, Quercetin 3-O-arabinoside, Cyanidin 3-O-(6″-acetyl-glucoside)		[17]

**Table 2:** Description of cell wall structure and composition of the main marine microalgae representatives.

Microalgae	Cell Wall Structure	Ref
*Isochrysis galbana*	It lacks a cell wall, which makes it fragile.	[75]
*Dunaliella salina*	It lacks a cell wall, having instead a thin elastic plasma membrane, and a glycocalyx surface coat that makes the cells morphologically more flexible.	[75,76,77,78]
*Porphyridium purpureum*	It lacks a cell wall. It secretes extracellular polysaccharides that surround the cell	[79]
*Phaeodactylum tricornutum*	Three-layer cell wall. Poor in silica. Mainly composed of sulphated glucuronomannan, proteins, and long-chain polyamines	[75,80,81]
*Tetraselmis*	The transition between scaled-wall and rigid-wall. The cell is surrounded by a rigid wall (theca). It secretes stellate scales that are fused extracellularly into a wall-like structure.	[82]
*Chlorella minutissima*	Thick cell wall (50–90 nm) with two distinct layers. The inner layer is a thick electron-dense microfibrillar sheet. The outer layer is trilaminate, comprising two dense electron sheets and an electron-translucent sublayer.	[82]
*Nannochloropsis* sp.	Bilayered cell-wall formed of an algaenan-containing rigid trilaminar layer structure cell wall as the external layer. The inner wall layer is composed of cellulose. The cell wall is mainly composed of mannan-type hemicelluloses.	[83,84]

**Table 3:** Mechanical cell disruption techniques applied to microalgae focusing on recovery studies of phenolic compounds.

Mechanical Cell Disruption Technique	Microalgae	Equipment	Treatment Parameters	Ref
Bead milling	*Phaeodactylum tricornutum* and *Tetraselmis chui*	Dyno-Mill Multi Lab bead mill	Grinding chamber: 0.6 L. Glass beads: 0.25–0.4 mm filled 85% of chamber volume. Agitator tip speed: 8–12 m/s (2391–3586 rpm for *P. tricornutum* and 1970–2852 rpm for *T. chui*). Biomass flow rate: 4–18 kg/h	[86]
	*Nannochloropsis* sp.	High-throughput ball mill homogenizer	6500 counts per minute (cpm) for 3 min with 15 s pauses each minute (repeated 8 times)	[68]
High-speed Homogenization	*Tetraselmis chuii*, *Nannochloropsis oculata*, *Chlorella minutissima* and *Rhodomonas salina*	Ultra-Turrax	Not reported	[34]
	*Amphidinium carterae*, *Coccolithophorid* sp., *Navicula* sp., *Dunaliella tertiolecta*, *Microchloropsis salina*, *Proteomonas sulcate*, and *Tetraselmis suecica*	Ultra-Turrax	Homogenized for 30 s	[17]
	*Phaeodactylum tricornutum* and *Pavlova lutheri*	Ultra-Turrax	14,000 rpm for 30 s	[87]
Pulsed Electric Field	*Nannochloropsis* sp.	High voltage pulsed power 40 kV–10 kA generator	*E:* 20 kV/cm, 1–4 ms, 13.3–53.1 kJ/kg. N = 400 pulses. The gap between electrodes was set at 2 cm. Δt = 2 s. T = 293 K	[48]
	*Phaeodactylum tricornutum* and *Tetraselmis chui*	PEF-Cellcrack III	(I) 1 kV/cm/400 pulses (II) 3 kV/cm/45 pulses, 100 kJ/kg 100 ms and 2 Hz The gap between electrodes was set at 10 cm	[70]
Ultrasonication	*Chlorella* sp.	Ultrasonic bath	20 kHz for 5 min	[88]
Microwave	*Spirulina* sp.	Microwave oven	2450 MHz, 1400 W, 120 s	[42]
	*Coelastrella* sp.	Conventional microwave	800 W, 12 min.	[65]
Freeze-thaw cycles	*Spirulina platensis*	Not reported	Rapid freezing (−20 °C) and thawing (4 °C) process repeated 4 times	[89]
	*Coelastrella* sp.	Hot water bath (for thawing)	Freezing (−80 °C) and thawing (40 °C) for 10 cycles	[65]

**Table 4:** Chemical cell disruption techniques applied on marine microalgae for the release of polyphenols.

Chemical Cell Disruption Technique	Microalgae	Phenolic Content. TPC = mg GAE/g DW; TFC = mg QE/g DW; TCT = mg CE/g DW	Ref
(I) Alkaline hydrolysis: 2M NaOH, 60 min (II) Acid hydrolysis: Concentrated HCl to pH = 2	*Proteomonas sulcata*	TPC (free) = 1.83 ± 0.16, TPC (bound) = 4.03 ± 0.20. TFC (free) = 0.24 ± 0.02, TFC (bound) = 0.43 ± 0.03. TCT (free) = 0.09, TCT (bound) = 11.36 ± 0.92. Identified: Gallic acid, 4′-O-methyl-(-)-epigallocatechin 7-O-glucuronide, Apigenin 6,8-di-C-glucoside, Quercetin 3′-sulfate, Secoisolariciresinol-sesquilignan, Schisandrin	[17]
	*Tetraselmis suecica*	TPC (free) = 4.72 ± 0.12, TPC (bound) = 11.67 ± 0.55. TFC (free) = 1.36 ± 0.04, TFC (bound) = 0.98 ± 0.10. Identified: Cyanidin 3-O-(6″-acetyl-glucoside), Scopoletin	
	*Amphidinium carterae*	TPC (free) = 6.45 ± 0.42, TPC (bound) = 26.03 ± 0.34. TCT (bound) = 15.22 ± 0.86 Identified: Apigenin 6,8-di-C-glucoside, Quercetin 3-O-arabinoside, Cyanidin 3-O-(6″-acetyl-glucoside), Schisandrin	
	*Coccolithophorid* sp.	TPC (free) = 3.76 ± 0.24, TPC (bound) = 19.63 ± 0.34. TFC (free) = 0.79 ± 0.09, TFC (bound) = 1.61 ± 0.15 TCT (bound) = 20.43 ± 0.16. Identified: Chrysoeriol 7-O-glucoside, Scopoletin, Schisandrin	
	*Dunaliella tertiolecta*	TPC (free) = 4.67 ± 0.35, TPC (bound) = 12.49 ± 0.21 TFC (free) = 1.03 ± 0.09, TFC (bound) = 0.54 ± 0.05. TCT (bound) = 1.78 ± 0.18. Identified: 4′-O-methyl-(-)-epigallocatechin 7-O-glucuronide,	
	*Nannochloropsis salina*	TPC (free) = 2.78 ± 0.22, TPC (bound) = 21.32 ± 0.65 TFC (free) = 0.17 ± 0.00, TFC (bound) = 1.66 ± 0.05 TCT(free) = 1.17 ± 0.16, TCT (bound) = 19.75 ± 1.41. Identified: 4′-O-methyl-(-)-epigallocatechin 7-O-glucuronide, Chrysoeriol 7-O-glucoside, Schisandrin	
	*Navicula* sp.	TPC (free) = 2.60 ± 0.08, TPC (bound) = 5.16 ± 0.34 TFC (free) = 0.04 ± 0.01, TFC (bound) = 0.35 ± 0.03. Identified: 4′-O-methyl-(-)-epigallocatechin 7-O-glucuronide, Apigenin 6,8-di-C-glucoside, Quercetin 3-O-arabinoside, Cyanidin 3-O-(6″-acetyl-glucoside)	

**Table 6:** Ultrasound-Assisted Extraction (UAE) methods developed to date for the optimal recovery of polyphenols from marine microalgae.

Microalgae	Pre-Treatment	Solvent(s)	UAE Parameters	Extraction Time × Cycles	Phenolic Content	Ref
*Chaetoceros* sp.	Freeze-dried	Water	S/L ratio (*w*/*v*): 1:25 (2 g in 50 mL). Ultrasonic probe: 20 kHz, 400 W	15 min × 1	Not reported	[28]
*Chlorella* sp.	Freeze-dried and milled	Methanol/water (80:20 and 50:50 *v*/*v*), ethanol/water (80:20 and 50:50 *v*/*v*)	S/L ratio (*w*/*v*): 1:20 (0.075 g in 1.5 mL). Ultrasonic bath (kHz: not reported, W: not reported).	30 min × 3	TPC = 6.13 mg GAE/g DW TFC = 7.38 mg CT/g DW	[33]
	Freeze-dried	Water	S/L ratio (*w*/*v*): 1:25 (2 g in 50 mL). Ultrasonic probe: 20 kHz, 400 W	15 min × 1	Not reported	[28]
*Diacronema lutheri*	Freeze-dried and ground using a pestle and mortar	Methanol	S/L ratio (*w*/*v*): 1:10 (0.5 g in 5 mL). Ultrasonic bath: 40 kHz, 100 W	15 min × 2	p-coumaric acid, ferulic acid, apigenin, quercetin, phloretin, naringenin, dihydroquercetin, dihydrokaempherol.	[18]
*Dunaniella salina*	Freeze-dried	Water	S/L ratio (*w*/*v*): 1:25 (2 g in 50 mL). Ultrasonic probe: 20 kHz, 400 W	15 min × 1	Not reported	[28]
	Freeze-dried and ground into a fine powder	Methanol	S/L ratio (*w*/*v*): 1:100 (0.05 g in 5 mL). Ultrasonic bath: 40 kHz (W: not reported)	45 min × 2	TPC = 4.52 mg GAE/g. Identified: caffeic acid, ferulic acid, p-coumaric acid.	[20]
*Dunaniella* sp.	Freeze-dried	Water	S/L ratio (*w*/*v*): 1:25 (2 g in 50 mL). Ultrasonic probe: 20 kHz, 400 W	15 min × 1	Not reported	[28]
*Isochrysis* sp.	Freeze-dried	Water	S/L ratio (*w*/*v*): 1:25 (2 g in 50 mL). Ultrasonic probe: 20 kHz, 400 W	15 min × 1	Not reported	[28]
*Nannochloris* sp.	Freeze-dried and ground to a fine powder	Methanol	S/L ratio (*w*/*v*): 1:10 (0.3 g in 3 mL). Ultrasonic bath (kHz: not reported, W: not reported).	45 min × 3	TPC = 33.23 mg GAE/g EW TFC = 4.22 mg QE/g DW. Identified: protocatechuic acid, caffeoyl coumaroyl-quinic acid, catechin, dimethoxyflavone, kaempferol;	[19]
*Nannochloropsis gaditana*	Freeze-dried and milled	Methanol/water (80:20 and 50:50 *v*/*v*), ethanol/water (80:20 and 50:50 *v*/*v*)	S/L ratio (*w*/*v*): 1:20 (0.075 g in 1.5 mL). Ultrasonic bath (kHz: not reported, W: not reported).	30 min × 3	TPC = 5.47 mg GAE/g DW TFC = 6.49 mg CT/g DW.	[33]
	Freeze-dried and ground to a fine powder	Methanol	S/L ratio (*w*/*v*): 1:10 (0.3 g in 3 mL). Ultrasonic bath (kHz: not reported, W: not reported).	45 min × 3	TPC = 22.94 mg GAE/g EW TFC = 5.18 mg QE/g DW. Identified: protocatechuic acid, caffeic acid, quercetin, caffeoyl glucoside, feruloylglucaric acid, p-coumaroyl tyrosine, apigenin-O-rutinoside, rhamnosyl hexosyl-methyl quercetin;	[19]
	Freeze-dried	Water	S/L ratio (*w*/*v*): 1:25 (2 g in 50 mL). Ultrasonic probe: 20 kHz, 400 W	15 min × 1	Not reported	[28]
*Nannochloropsis oculata*	Freeze-dried	Methanol:water:acetic acid:ascorbic acid (30:67:1:2, *v*/*v*/*v*/*w*)	S/L ratio (*w*/*v*): 1:70 (0.1 g in 7 mL). Ultrasonic bath: 40 kHz, 300 W	15 min × 2	Flavan-3-ols = 174.65 mg/100g DW Phenolic acids = 22.08 mg/100g DW	[29]
*Nannochloropsis salina*	Freeze-dried and ground into a fine powder	Methanol	S/L ratio (*w*/*v*): 1:100 (0.05 g in 5 mL). Ultrasonic bath: 40 kHz (W: not reported)	45 min × 2	TPC = 6.45 mg GAE/g. Identified: Gallic acid, 3,4 dihydroxy benzoic acid, ferulic acid, p-coumaric acid, salicylic acid.	[20]
*Nannochloropsis* sp.	Frozen paste thawed at ambient temperature and diluted with solvents	Water, ethanol, DMSO and their mixture (water/DMSO and water/ethanol)	S/L ratio (*w*/*v*): 1:10. Ultrasonic probe: 24 kHz, 400 W	5 min × 1	Maximum recovery of phenolic compounds using water-DMSO and water-EtOH at 25–30%.	[68]
*Navicula* sp.	Freeze-dried	Water	S/L ratio (*w*/*v*): 1:25 (2 g in 50 mL). Ultrasonic probe: 20 kHz, 400 W	15 min × 1	Not reported	[28]
*Naviculoid diatom* (*Strain IMA053*)	Freeze-dried	Water, methanol, dichloromethane (DCM)	S/L ratio (*w*/*v*): 1:200 (1 g in 200 mL). Ultrasonic bath: 45 kHz, 400 W	15 min × 3	TPC = 17.80 (water), 31.85 (methanol), 40.58 (DCM) mg GAE/100 g EW	[35]
*Phaeodactylum tricornutum*	Freeze-dried and ground to a fine powder	Methanol	S/L ratio (*w*/*v*): 1:10 (0.3 g in 3 mL). Ultrasonic bath (kHz: not reported, W: not reported).	45 min × 3	TPC = 39.34 mg GAE/g EW TFC = 3.05 mg QE/g DW. Identified: protocatechuic acid, caffeic acid, caffeic acid hexoside dimer, dimethoxyflavone, p-coumaroyl tyrosine	[19]
	Freeze-dried	Water	S/L ratio (*w*/*v*): 1:25 (2 g in 50 mL). Ultrasonic probe: 20 kHz, 400 W	15 min × 1	Not reported	[28]
	Freeze-dried and ground using a pestle and mortar	Methanol	S/L ratio (*w*/*v*): 1:10 (0.5 g in 5 mL). Ultrasonic bath: 40 kHz, 100 W	15 min × 2	p-coumaric acid, ferulic acid, daidzein and genistein.	[18]
	Freeze-dried and ground into a fine powder	Methanol	S/L ratio (*w*/*v*): 1:100 (0.05 g in 5 mL). Ultrasonic bath: 40 kHz (W: not reported)	45 min × 2	TPC = 3.16 mg GAE/g. Identified: 3,4 dihydroxy benzoic acid, p-coumaric acid, salicylic acid.	[20]
*Porphyridium purpureum*	Freeze-dried	Methanol:water:acetic acid:ascorbic acid (30:67:1:2, *v*/*v*/*v*/*w*)	S/L ratio (*w*/*v*): 1:70 (0.1 g in 7 mL). Ultrasonic bath: 40 kHz, 300 W	15 min × 2	Flavan-3-ols = 207.30 mg/100 g DW.	[29]
	Freeze-dried and ground using a pestle and mortar	Methanol	S/L ratio (*w*/*v*): 1:10 (0.5 g in 5 mL). Ultrasonic bath: 40 kHz, 100 W	15 min × 2	p-coumaric acid, ferulic acid, apigenin, luteolin, daidzein, genistein, quercetin.	[18]
*Tetraselmis* sp.	Freeze-dried	Water	S/L ratio (*w*/*v*): 1:25 (2 g in 50 mL). Ultrasonic probe: 20 kHz, 400 W	15 min × 1	Not reported	[28]
*Tetraselmis suecica*	Freeze-dried and ground to a fine powder	Methanol	S/L ratio (*w*/*v*): 1:10 (0.3 g in 3 mL). Ultrasonic bath (kHz: not reported, W: not reported).	45 min × 3	TPC = 28.03 mg GAE/g EW TFC = 0.61 mg QE/g DW. Identified: protocatechuic acid, caffeic acid, caffeoyl glucoside, dimethoxyflavone, p-coumaroyl tyrosine, apigenin-O-rutinoside, rhamnosyl hexosyl-methyl-quercetin	[19]
	Freeze-dried and ground using a pestle and mortar	Methanol	S/L ratio (*w*/*v*): 1:10 (0.5 g in 5 mL). Ultrasonic bath: 40 kHz, 100 W	15 min × 2	p-coumaric acid, ferulic acid, apigenin, daidzein,	[18]
*Tetraselmis marina* (*Strain IMA043*)	Freeze-dried	Water, methanol, dichloromethane (DCM)	S/L ratio (*w*/*v*): 1:200 (1 g in 200 mL). Ultrasonic bath: 45 kHz, 400 W	15 min × 3	TPC = 20.45 (water), 25.19 (methanol), 86.14 (DCM) mg GAE/100 g EW	[35]

**Table 7:** Microwave-Assisted Extraction (MAE) methods developed to date for the optimal recovery of polyphenols from marine microalgae.

Microalgae	Pre-Treatment	Solvent(s)	MAE Parameters	Extraction Time	Phenolic Content	Ref
*Phaeodactylum tricornutum*	Cell disruption at high pressure (1200 bar) and then freeze-dried	Ethanol 100%	S/L ratio (*w*/*v*): 1:20 (0.5 g in 10 mL) Temperature: 30 °C Frequency: 2.45 GHz	2 min	46.57 mg GAE/g EW	[105]

**Table 8:** Pressurized Liquid Extraction (PLE) methods developed to date for the optimal recovery of polyphenols from marine microalgae.

Microalgae	Pre-Treatment	Solvent(s)	PLE Parameters	Extraction Time	Phenolic Content	Ref
*Chlorella*	Freeze-dried	100% DMSO	Microalgae-Solid ratio: 0.5 g in 1.5 g of diatomaceous earth. Cell volume: not reported. Temperature: 40 °C. Pressure: 103.4 bar	15 min	10 mg GAE/g DW	[52]
*Phaeodactylum tricornutum*	Freeze-dried	100% DMSO	Microalgae-Solid ratio: 0.5 g in 1.5 g of diatomaceous earth. Cell volume: not reported. Temperature: 40 °C. Pressure: 103.4 bar	15 min	11.5 mg GAE/g DW	[52]
	Cell disruption at high pressure (1200 bar) and then freeze-dried	100% Ethanol	Microalgae-Solid ratio: 1 g in 2.5 g of sea sand. Cell volume: 11 mL. Temperature: 50 °C. Pressure: 100 bar	20 min	42.16 mg GAE/g EW (10.14 mg GAE/g DW)	[105]
*Tetraselmis chuii*	Not reported	50% ethyl acetate in cyclopentyl methyl ether	Microalgae-Solid ratio: 1 g between two layers of sea sand (2 g each). Cell volume: 11 mL. Temperature: 180 °C. Pressure: 105 bar	20 min	644.52 mg GAE/g EW (73.28 mg GAE/g DW)	[107]

**Table 9:** Supercritical CO_2_ Extraction (SC-CO_2_) of polyphenols from marine microalgae.

Microalgae	Pre-Treatment	Solvent(s)	SC-CO_2_ Parameters	Extraction Time	Phenolic Content	Ref
*Nannopclorosis gaditana*	Freeze-dried	CO_2_ + Ethanol (5%)	CO_2_ flow rate: 20 g/min. Temperature: 55 °C. Pressure: 100 bar	2 h	≈5 mg GAE/g EW	[135]
*Tetraselmis chuii*	Spray-dried and pre-treated with alumina oxide (1:1 *w*/*w*) for 3 h	CO_2_ + Ethanol (20%)	CO_2_ flow rate: 20 g/min. Temperature: 55 °C. Pressure: 100 bar	2 h	2.5 mg GAE/g EW	[135]

**Table 10:** TPC values and LC-MS/MS polyphenols determination of different extracts from *Porphyridium cruentum* [148]. * Data derive from the sum of all the determined polyphenols.

Analytical Technique	Soxhlet, 96% Ethanol	Soxhlet, Ethanol/n-Hexane	400 Bar, 40 °C, 10% Ethanol
TPC (Quercetin equivalent μg mg^−1^)	134.40 ± 0.80	162.45 ± 3.40	182.09 ± 8.08
LC–MS/MS (ng mg^−1^)	78.48 *	53.73 *	58.25 *

**Table 14:** Identified polyphenols in marine microalgae species in comparison with different analytical methods.

	MICROALGAE															
	*Nannochloropsis* sp.		*Nannochloropsis oculata*			*Phaeodactylum tricornutum*			*Porphyridium purpureum*		*Spirulina maxima*		*Tetraselmis suecica*			*Tisochrysis lutea*
Method of Analysis →	HPLC-DAD [193]	HPLC-DAD-MS [19]	HPLC-UV-Vis [44]	UHPLC-DAD [29]	LC-MS [204]	HPLC-DAD [20]	HPLC-DAD-MS [19]	UHPLC-MS/MS [18]	UHPLC-DAD [29]	UHPLC-MS/MS [18]	HPLC-DAD [192]	GC-FID [40]	HPLC-DAD-MS [19]	UHPLC-MS/MS [18]	LC-QTOF-MS/MS [17]	HPLC-DAD [193]
Polyphenols ↓																
Hydroxybenzoic acid and/or derivatives																
Apigenin and/or derivatives																
Caffeic acid and/or derivatives																
Caffeoyl-coumaroyl-quinic acid																
Catechin																
Chicoric acid																
Chlorogenic acid																
Chrysin																
Cinnamic acid																
Cyanidin 3-O-(6″-acetyl-glucoside)																
Daidzein																
Dimethoxyflavone																
Epicatechin and/or derivatives																
Eriodictyol																
Eugenol																
Ferulic acid																
Galangin																
Gallic acid																
Genistein																
Kaempferol and/or derivatives																
Luteolin and/or derivatives																
Morin																
Naringenin																
p-coumaric acid and/or derivatives																
p-coumaroyl tyrosine																
Phloretin																
Phloroglucinol																
Pinostrobin																
Procyanidin A2																
Protocatechuic acid																
Quercetin and/or derivatives																
Quinic acid																
Rutin																
Salicylic acid																
Scopoletin																
Synaptic acid																
Syringic acid																
Vanillic acid																
Vitexin																

**Table 15:** Marine microalgae species with their origin and used solvents for the polyphenolic extracts.

Microalgae	Method of Analysis	Microalgae Origin	Solvent of Extraction	Ref
*Nannochloropsis* sp.	HPLC-DAD	Purchased: Proviron company (Hemiksem, Belgium)	methanol	[193]
	HPLC-DAD-MS	Cultured: air stream 5% of CO_2_, 16 fluorescent lamps, 21 °C.	methanol/acetone	[19]
*Nannochloropsis oculata*	HPLC-UV-Vis	Cultivated: f/2 medium for 45 days (Guillard 1975)	methanol	[44]
	UHPLC-DAD	Cultivated: Department of Biosciences of Swansea University	methanol/water/acetic acid/ascorbic acid	[29]
	LC-MS	Cultivated: adapted method Sharifah et al.	methanol	[204]
*Phaeodactylum tricornutum*	HPLC-DAD	Cultivated: National Food Institute, Schott bottles, 2% carbon dioxide/air, fluorescent lights 300 µ mol photon m^−2^·s^−1^	methanol	[20]
	HPLC-DAD-MS	Cultured: air stream 5% of CO_2_, 16 fluorescent lamps, 21 °C.	methanol	[19]
	UHPLC-MS/MS	Cultivated: synthetic seawater, 30 g L^−1^ synthetic sea salt in deionized water (Homarsel, Zoutman, Belgium)	methanol	[18]
*Porphyridium purpureum*	UHPLC-DAD	Cultivated: Department of Biosciences of Swansea University (ScanVac Cool Safe, LaboGene; Lynge, Denmark)	methanol/water/acetic acid/ascorbic acid	[29]
	UHPLC-MS/MS	Cultivated: synthetic seawater, 30 g L^−1^ synthetic sea salt in deionized water (Homarsel, Zoutman, Belgium)	methanol	[18]
*Spirulina maxima*	HPLC-DAD	Culture Collection of Texas University, Austin, TX, USA	ethanol	[192]
	GC-FID	Cultivated: Paoletti’s culture medium, 5000 lux, 14 h light and 10 h dark, 20 °C. Oceanographic Institute of the University of São Paulo (Brazil)	methanol	[40]
*Tetraselmis suecica*	HPLC-DAD-MS	Cultured: air stream 5% of CO_2_, 16 fluorescent lamps, 21 °C.	methanol	[19]
	UHPLC-MS/MS	Cultivated: synthetic seawater, 30 g L^−1^ synthetic sea salt in deionized water (Homarsel, Zoutman, Belgium)	methanol	[18]
	LC-QTOF-MS/MS	Culture collection Department of Chemical Engineering, University of Melbourne	ethanol	[17]
*Tisochrysis lutea*	HPLC-DAD	Purchased: Proviron company (Hemiksem, Belgium)	methanol	[193]
